# Injury mortality and morbidity changes due to the COVID-19 pandemic in the United States

**DOI:** 10.3389/fpubh.2022.1001567

**Published:** 2022-11-02

**Authors:** Jieyi He, Peishan Ning, David C. Schwebel, Yang Yang, Li Li, Peixia Cheng, Zhenzhen Rao, Guoqing Hu

**Affiliations:** ^1^Department of Epidemiology and Health Statistics, Hunan Provincial Key Laboratory of Clinical Epidemiology, Xiangya School of Public Health, Central South University, Changsha, China; ^2^Department of Psychology, University of Alabama at Birmingham, Birmingham, AL, United States; ^3^Department of Statistics, Franklin College of Arts and Sciences, University of Georgia, Athens, GA, United States; ^4^National Clinical Research Center for Geriatric Disorders, Xiangya Hospital, Central South University, Changsha, China

**Keywords:** injury, mortality, morbidity, COVID-19 pandemic, United States

## Abstract

**Introduction:**

The COVID-19 pandemic significantly changed society. We aimed to examine the systematic impact of the COVID-19 on injury burden in the United States.

**Methods:**

We extracted mortality and morbidity data from CDC WONDER and WISQARS. We estimated age-standardized injury mortality rate ratio and morbidity rate ratio (MtRR and MbRR) with 95% confidence interval (95% CI) for all injuries, all unintentional injuries, homicide/assault by all methods, suicide/self-harm by all methods, as well as other 11 specific unintentional or intentional injury categories. Injury rate ratios were compared for 2020 vs. 2019 to those of 2019 vs. 2018 to demonstrate the influence of the COVID-19 pandemic on fatal and nonfatal injury burden. The ratio of MtRRs (RMtRR) and the ratio of MbRRs (RMbRR) with 95% CI between 2020 vs. 2019 and 2019 vs. 2018 were calculated separately.

**Results:**

The COVID-19 pandemic was associated with an increase in injury mortality (RMtRR = 1.12, 95% CI: 1.11, 1.13) but injury morbidity decreased (RMbRR = 0.88, 95% CI: 0.88, 0.89) when the changes of these rates from 2019 to 2020 were compared to those from 2018 to 2019. Mortality disparities between the two time periods were primarily driven by greater mortality during the COVID-influenced 2020 vs. 2019 from road traffic crashes (particularly motorcyclist mortality), drug poisoning, and homicide by firearm. Similar patterns were not present from 2019 vs. 2018. There were morbidity reductions from road traffic crashes (particularly occupant and pedestrian morbidity from motor vehicle crashes), unintentional falls, and self-harm by suffocation from 2019 to 2020 compared to the previous period. Change patterns in sexes and age groups were generally similar, but exceptions were observed for some injury types.

**Conclusions:**

The COVID-19 pandemic significantly changed specific injury burden in the United States. Some discrepancies also existed across sex and age groups, meriting attention of injury researchers and policymakers to tailor injury prevention strategies to particular populations and the environmental contexts citizens face.

## Introduction

The COVID-19 pandemic has lasted over 2 years and impacted the world greatly, killing 6.06 million persons directly by March 18, 2022 (1) and increasing the risk of many other diseases and injuries through pandemic-associated increased stress, changing transportation modes, reduced access to or use of healthcare services, and other behavior changes (2–5).

Researchers and policymakers can benefit from evaluation of the impact of the COVID-19 pandemic on other diseases and injuries to prepare for future COVID-19-related outbreaks or other public health crises. Early data suggest injury rates, which represent a major global health problem, were affected substantially by the COVID-19 pandemic. Published studies using hospital admissions (6–9), social media data (10), and online self-reports (11) to examine the adverse consequences of the COVID-19 pandemic on suicide/self-poisoning (6, 7), domestic injuries (9), and violence (8, 10, 11) report decreased hospital admissions for suicide/self-poisoning and increased domestic violence and home injury during the pandemic. Several other studies used governmental surveillance databases to assess changes in mortality from suicide, road traffic crashes, or falls in specific countries and detected inconsistent injury mortality changes across cause of injury and countries. Reports include decreases in road traffic mortality and increases in unintentional fall mortality in China (12), as well as inconsistent suicide mortality changes across countries [increases in China (12) and Japan (13) but unchanged suicide rates in Queensland, Australia (14) and Massachusetts, USA] (15) in 2020 compared to 2019. All these studies, however, only examined changes in morbidity or mortality from individual types of injuries. Many were conducted during the early stages of the COVID-19 pandemic. Currently, no published studies assess the systematic impact of the COVID-19 pandemic on all types of injury morbidity and mortality in any single country.

The United States is one of the countries globally that regularly collects and publicly releases its injury morbidity and mortality data to the public (16, 17). Using recently released data, we compared changes in both mortality and morbidity between 2020 vs. 2019 and 2019 vs. 2018 to examine the influence of the COVID-19 pandemic on the injury burden in the United States.

## Materials and methods

### Data sources

Injury mortality data were extracted from the underlying cause of death public use data in the CDC Wide-ranging Online Data for Epidemiologic Research (CDC WONDER) system (16). Mortality data are based on information from all death certificates filed in the fifty states and the District of Columbia spanning the years 1999 to 2020 (17).

Injury morbidity data were extracted from the CDC's Web-based Injury Statistics Query and Reporting System (CDC's WISQARS) (18). Injury morbidity data are based on all causes of non-fatal injuries and poisonings treated in U.S. hospital emergency departments (EDs) and are collected by the National Electronic Injury Surveillance System-All Injury Program (NEISS-AIP). The NEISS-AIP is a nationally representative sample consisting of 66 of the 100 NEISS hospitals since 2000, covering very large inner-city hospitals with trauma centers as well as large urban, suburban, rural, and children's hospital (19).

Based on our preliminary analyses (not shown here), we extracted data from the two data sources mentioned above and selected 15 injury categories with high mortality rates to guide subsequent analyses (Table 1).

**Table 1:** ICD-10 codes of the 15 selected injury types.

**Cause of injury**	**ICD-10 code**
All injuries	(V01–Y36, Y85–Y87, Y89)
Unintentional injuries	(V01–X59, Y85–Y86)
Motor vehicle traffic	[V30–V39 (0.4–0.9), V40–V49 (0.4–0.9), V50–V59 (0.4–0.9), V60–V69 (0.4–0.9), V70–V79 (0.4–0.9), V81.1, V82.1, V83–V86 (0.0–0.3), V20–V28 (0.3–0.9), V29 (0.4–0.9), V12–V14 (0.3–0.9), V19 (0.4–0.6), V02–V04 (0.1, 0.9), V09.2, V80 (0.3–0.5), V87 (0.0–0.8), V89.2]
Occupant	[V30–V39 (0.4–0.9), V40–V49 (0.4–0.9), V50–V59 (0.4–0.9), V60–V69 (0.4–0.9), V70–V79 (0.4–0.9), V83–V86 (0.0–0.3)]
Motorcyclist	[V20–V28 (0.3–0.9), V29 (0.4–0.9)]
Bicyclist	[V12–V14 (0.3–0.9), V19 (0.4–0.6)]
Pedestrian	[V02–V04 (0.1, 0.9), V09.2]
Falls	(W00–W19)
Poisoning	(X40–X49)
Drug poisoning	(X40–X44)
Homicide/assault	(X85–Y09, Y87.1)
By firearm	(X93–X95)
Suicide/self–harm	(X60–X84, Y87.0)
By firearm	(X72–X74)
By suffocation	(X70)

### Statistical analysis

Injury mortality and morbidity rates were age-standardized using the national projected population of 2000 as the standard population. Following the United States CDC's recommendation (17, 19), we did not report unstable mortality or morbidity rates that were calculated based on fewer than 20 deaths or 20 injuries.

Preliminary analyses (not shown here) indicated that both injury mortality and morbidity rates were stable from 2010 to 2019, but many injuries categories demonstrated dramatic changes between 2019 and 2020. Following the theory of difference-in-differences method, we compared the mortality rate ratio (MtRR) and morbidity rate ratio (MbRR) of 2020 vs. 2019 (during the pandemic) to those of 2019 vs. 2018 (before the pandemic) to examine the possible influence of the COVID-19 pandemic on fatal and nonfatal injury burden. The difference-in-differences method controls for long-term trends in mortality and morbidity unrelated to the COVID-19 pandemic, such as population aging and improvements in injury prevention technologies and policies. Using binomial regression models with a log link, we calculated the ratio of mortality rate ratios (RMtRR) and the ratio of morbidity rate ratios (RMbRR) with 95% confidence interval (95% CI) between 2020 vs. 2019 and 2019 vs. 2018 and tested their statistical significance at the 0.05 level to reflect the likely impact of the COVID-19 pandemic. The MtRRs, MbRRs, and relevant 95% CIs were calculated by the Woolf method. Bar charts were plotted to demonstrate differences in age-standardized mortality/morbidity rate ratio with 95% CI between 2020 vs. 2019 and 2019 vs. 2018.

Considering the large injury mortality and morbidity disparities across sex and age groups, we also performed subgroup analyses by sex (male, female) and age group (0–24 years old, 25–44 years old, 45–64 years old, and 65 years and older).

All statistical analyses were performed using PASW Statistics 18.0.0 and R version 4.1.2.

### Ethics concerns

Injury mortality and morbidity data were publicly available and did not involve identifiable information. Our analysis strictly complied with all data use restrictions terms for the online databases and was exempt from IRB review at our university.

## Results

**Figure 1:**
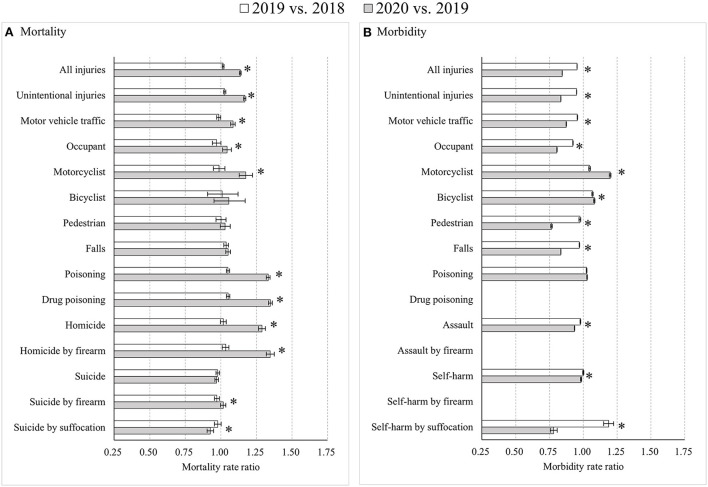
Age-standardized injury mortality/morbidity rate ratios among Americans for all age groups, 2019 vs. 2018 and 2020 vs. 2019. **(A)** Mortality; **(B)** Morbidity. Results were omitted for categories having unstable injury morbidity rates (due to the computation based on cases <20, the national estimates <1,200, the coefficient of variation > 30%, or the tool not involving details regarding mechanism of relevant injury). “*” indicates that the differences of MtRRs or MbRRs between 2020 vs. 2019 and 2019 vs. 2018 were statistically significant.

The COVID-19 pandemic led to distinct changes in injury mortality and injury morbidity during the time period from 2018 to 2020. Overall age-standardized injury mortality showed more increases from 2019 to 2020 (MtRR = 1.14) than from 2018 to 2019 (MtRR = 1.02) (with an RMtRR of 1.12, 95% CI: 1.11, 1.13), while overall injury morbidity decreased more from 2019 to 2020 (MbRR = 0.84) than from 2018 to 2019 (MbRR = 0.95) (with an RMbRR of 0.88, 95% CI: 0.88, 0.89) (Figure 1). The different mortality increases between the two time periods were primarily driven by greater mortality increases from road traffic crashes for motorcyclists, drug poisoning, and homicide by firearm during the pandemic than before the pandemic (Figure 1A). The accelerated decreases in morbidity between the time periods were mainly derived from substantial reductions in morbidity from occupants and pedestrians due to motor vehicle crashes, falls, and self-harm by suffocation during the pandemic (Figure 1B).

Sex-specific analyses showed very similar results for males and females, with two exceptions. First, increases in road traffic mortality during 2019–2020 tended to be more notable among males. Second, morbidity rates for females showed more decreases from 2019 to 2020 compared to from 2018 to 2019 for motor vehicle occupants (MbRR: 0.77 vs. 0.91; RMbRR: 0.850, 95% CI: 0.847, 0.854) and pedestrians (MbRR: 0.69 vs. 1.01; RMbRR: 0.69, 95% CI: 0.68, 0.70), as well as greater increases for motorcyclists (MbRR: 1.33 vs. 1.03; RMbRR: 1.29, 95% CI: 1.26, 1.33) and bicyclists (MbRR: 1.25 vs. 1.05; RMbRR: 1.19, 95% CI: 1.17, 1.21) (Supplementary Figure 1).

**Figure 2:**
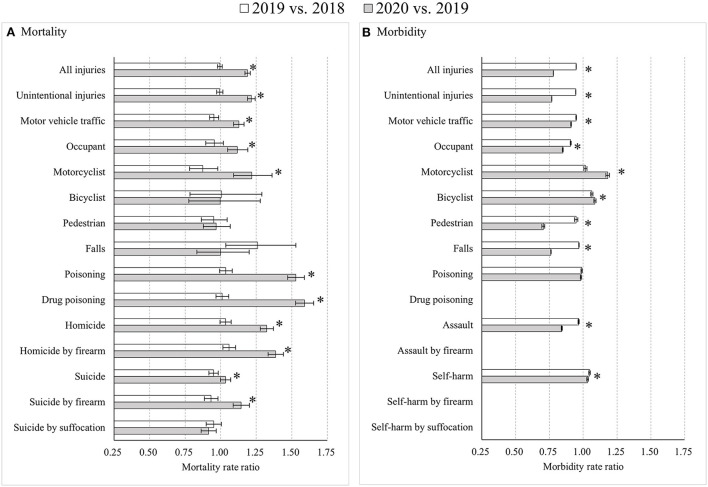
Age-standardized injury mortality/morbidity rate ratios among Americans aged 0–24 years old, 2019 vs. 2018 and 2020 vs. 2019. **(A)** Mortality; **(B)** Morbidity. Results were omitted for categories having unstable injury morbidity rates (due to the computation based on cases <20, the national estimates <1,200, the coefficient of variation >30%, or the tool not involving details regarding mechanism of relevant injury). “*” indicates that the differences of MtRRs or MbRRs between 2020 vs. 2019 and 2019 vs. 2018 were statistically significant.

Age-specific analyses also presented fairly similar results across groups, with several meaningful exceptions. Compared to the whole population, major differences in injury rates were found among the youngest age group of 0–24 years. First, road traffic mortality increased from 2019 to 2020, primarily among male motor vehicle occupants (*p* < 0.05). Second, unlike among other age groups, drug poisoning mortality differences between 2020 and 2019 were larger than differences between 2019 and 2018 for both males (MtRR: 1.62 vs. 1.05; RMtRR: 1.54, 95% CI: 1.41, 1.68) and females (MtRR: 1.47 vs. 0.99; RMtRR: 1.48, 95% CI: 1.29, 1.70). Third, suicide by firearm trended from decreases during 2018–2019 to increases during 2019–2020 for both males (MtRR: 1.14 vs. 0.95; RMtRR: 1.21, 95% CI: 1.10, 1.32) and females (MtRR: 1.19 vs. 0.89; RMtRR: 1.33, 95% CI: 1.03, 1.74). Last, assault morbidity showed greater decreases from 2019 to 2020 than from 2018 to 2019 for both males (MbRR: 0.82 vs. 0.98; RMbRR: 0.833, 95% CI: 0.825, 0.841) and females (MbRR: 0.87 vs. 0.95; RMbRR: 0.92, 95% CI: 0.91, 0.93) (Figure 2; Supplementary Figure 2).

**Figure 3:**
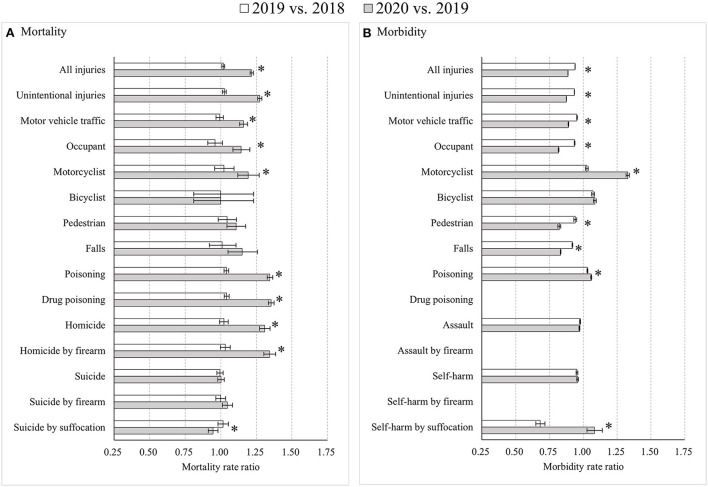
Age-standardized injury mortality/morbidity rate ratios among Americans aged 25–44 years old, 2019 vs. 2018 and 2020 vs. 2019. **(A)** Mortality; **(B)** Morbidity. Results were omitted for categories having unstable injury morbidity rates (due to the computation based on cases <20, the national estimates <1,200, the coefficient of variation >30%, or the tool not involving details regarding mechanism of relevant injury). “*” indicates that the differences of MtRRs or MbRRs between 2020 vs. 2019 and 2019 vs. 2018 were statistically significant.

For adults aged 25–44 years, notable differences compared to other age groups included four points. First, while stable during 2018–2019 for both sexes, mortality rates for motor vehicle occupants, motorcyclists and pedestrians all increased significantly from 2019 to 2020 among males (*p* < 0.05). Second, unintentional fall mortality increased significantly only among females from 2019 to 2020 (*p* < 0.05). Third, suicide mortality by firearm among males was flat during 2018–2019 (MtRR: 1.01, 95% CI: 0.97, 1.04) but jumped from 2019 to 2020 (MtRR: 1.04, 95% CI: 1.01, 1.08). Finally, self-harm morbidity among males decreased during 2018–2019 (MbRR: 0.88, 95% CI: 0.87, 0.89) but increased during 2019–2020 (MbRR: 1.02, 95% CI: 1.01, 1.03), with an RMbRR of 1.16 (95% CI: 1.14, 1.18). A slightly increasing trend was first seen among females during 2018–2019 (MbRR: 1.03, 95% CI: 1.02, 1.04) but followed by a substantial decrease during 2019–2020 (MbRR: 0.90, 95% CI: 0.89, 0.91), with an RMbRR of 0.88 (95% CI: 0.87, 0.89) (Figure 3; Supplementary Figure 3).

**Figure 4:**
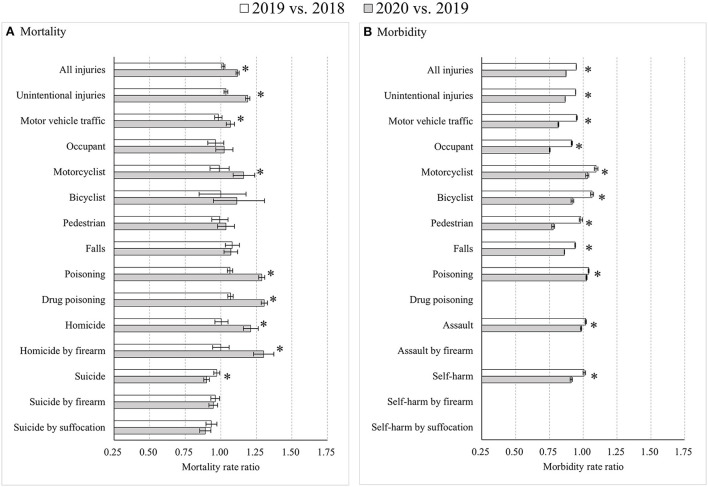
Age-standardized injury mortality/morbidity rate ratios among Americans aged 45–64 years old, 2019 vs. 2018 and 2020 vs. 2019. **(A)** Mortality; **(B)** Morbidity. Results were omitted for categories having unstable injury morbidity rates (due to the computation based on cases <20, the national estimates <1,200, the coefficient of variation >30%, or the tool not involving details regarding mechanism of relevant injury). “*” indicates that the differences of MtRRs or MbRRs between 2020 vs. 2019 and 2019 vs. 2018 were statistically significant.

For adults aged 45–64 years, notable differences compared to other age groups were detected. First, the changes in road traffic mortality rates were insignificant for all road users of both sexes from 2018 to 2019 (*p* > 0.05), but male motorcyclist mortality increased significantly from 2019 to 2020 (MtRR: 1.18, 95% CI: 1.10, 1.27), as did male bicyclist mortality (MtRR: 1.24, 95% CI: 1.05, 1.48) and male pedestrian mortality (MtRR: 1.09, 95% CI: 1.02, 1.17). Second, greater decreases in suicide mortality rates occurred among females than among males during both 2018–2019 and 2019–2020. Third, among males, road traffic morbidity rates increased for motorcyclists (MbRR: 1.14, 95% CI: 1.12, 1.15) and bicyclists (MbRR: 1.07, 95% CI: 1.06, 1.08) from 2018 to 2019, but decreased from 2019 to 2020 with an MbRR of 0.96 (95% CI: 0.95, 0.97) for motorcyclists and 0.88 (95% CI: 0.87, 0.89) for bicyclists. In contrast, morbidity in female motorcyclists decreased from 2018 to 2019 (MbRR: 0.88, 95% CI: 0.86, 0.91) but increased substantially from 2019 to 2020 (MbRR: 1.49, 95% CI: 1.45, 1.53). Fourth, the trend for self-harm morbidity among males reversed from an increase during 2018–2019 (MbRR: 1.18, 95% CI: 1.17, 1.20) to a decrease during 2019–2020 (MbRR: 0.87, 95% CI: 0.86, 0.88), with an RMbRR of 0.73 (95% CI: 0.72, 0.75), but self-harm morbidity among females continued to decline during 2019–2020 (MbRR: 0.96, 95% CI: 0.95, 0.98), though at a slower pace compared to 2018–2019 (MbRR: 0.86, 95% CI: 0.85, 0.87), with an RMbRR of 1.12 (95% CI: 1.09, 1.14) (Figure 4; Supplementary Figure 4).

**Figure 5:**
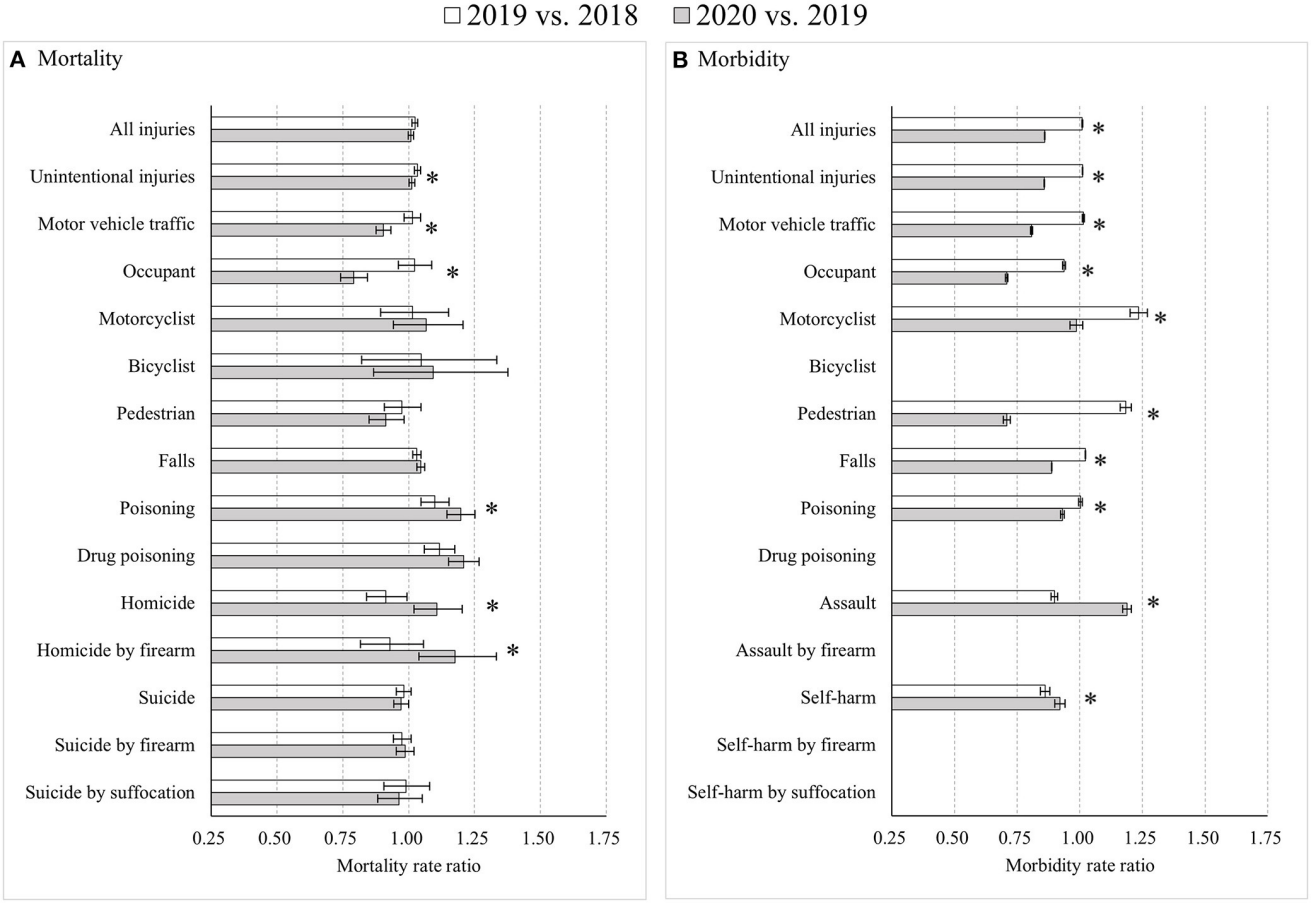
Age-standardized injury mortality/morbidity rate ratios among Americans aged 65 years and older, 2019 vs. 2018 and 2020 vs. 2019. **(A)** Mortality; **(B)** Morbidity. Results were omitted for categories having unstable injury morbidity rates (due to the computation based on cases <20, the national estimates <1,200, the coefficient of variation >30%, or the tool not involving details regarding mechanism of relevant injury). “*” indicates that the differences of MtRRs or MbRRs between 2020 vs. 2019 and 2019 vs. 2018 were statistically significant.

For older adults aged 65 years and older, unique patterns compared to other age groups were observed. First, the increase of overall injury mortality did not differ significantly between 2018–2019 and 2019–2020 (RMtRR: 0.985, 95% CI: 0.968, 1.003). Second, unlike the clear increases in road traffic mortality rates during 2019–2020 in other age groups, road traffic mortality among older adults declined substantially from 2019 to 2020 (MtRR: 0.90, 95% CI: 0.88, 0.93). Third, morbidity decreased significantly from 2019 to 2020 among male motorcyclists (MbRR: 0.96, 95% CI: 0.94, 0.99) and among both male (MbRR: 0.81, 95% CI: 0.79, 0.83) and female pedestrians (MbRR: 0.60, 95% CI: 0.59, 0.62), contrary to the substantial increases from 2018 to 2019. Fourth, the trend in assault morbidity reversed during 2019–2020, compared to 2018–2019, for both males (MbRR: from 0.88 to 1.18; RMbRR: 1.35, 95% CI: 1.31, 1.40) and females (MbRR: from 0.93 to 1.19, RMbRR: 1.29, 95% CI: 1.23, 1.34). Lastly, between 2018–2019 and 2019–2020, self-harm morbidity rate ratios increased among males (MbRR: from 0.68 to 1.25; RMbRR: 1.84, 95% CI: 1.74, 1.94) but decreased among females (MbRR: from 1.07 to 0.69; RMbRR: 0.65, 95% CI: 0.62, 0.68) (Figure 5; Supplementary Figure 5).

## Discussion

### Primary findings

Using the latest updated data, this study presents detailed evidence concerning the impact of the COVID-19 pandemic on injury burden in the United States between 2018 and 2020. Several key findings were generated. First, the overall age-standardized injury mortality rate increased more between 2019 and 2020 than between 2018 and 2019 but the overall injury morbidity rate decreased faster during the pandemic year of 2020. Second, the conflicting changes in injury mortality vs. morbidity were primarily driven by aggravated mortality increases from road traffic crashes (especially motorcyclists), drug poisoning, and homicide by firearm, and accelerated morbidity reductions in road traffic crashes (especially motor vehicle occupant and pedestrian morbidity), unintentional falls, and self-harm by suffocation. Third, we detected differing impacts of the pandemic on injury rates across sex and age groups.

### Interpretation of findings

The pattern of results we detected likely reflect the convergence of several contributing factors. Road traffic injury rates were likely affected by changes in exposure to certain road traveling modes due to social distancing restrictions during the pandemic (20) and economic factors changed traveling and community patterns (21), leading to reduced driving and increased riding and walking (22) as well as somewhat weakened injury prevention efforts (23). These patterns may have been especially true among older adults, who were least likely to travel outside the home, fearing COVID-19 infection, and their road traffic injury rates declined particularly drastically in 2020. There may also have been an increase in motorcycling or driving at high speeds to take advantage of emptier roadways associated with reduced presence of law enforcement (24), dangerous behaviors that increased mortality especially among young men. Parallel findings were previously reported from United Arab Emirates (25) and Virginia, U.S. (26).

The decrease in fall-related injuries was also likely influenced by lockdown measures and stay-at-home orders. These factors were likely to impact two age groups in particular, children/teens and older adults. Children and teens likely saw a decrease in falls during recreational activities such as sports and playground play while older adults likely saw a decrease in falls while exercising, shopping, or otherwise engaging in activities outside the home. Simultaneously, because people spent more time at home, it was reasonable to see morbidity increases for indoor injury causes during the pandemic, such as hospital admissions after indoor falls (9).

The substantial increase in poisoning mortality may be associated with drug overdoses among people who were lonely, bored, or suffering from mental disorders exacerbated by economic and emotional stresses of the pandemic (21, 27). The increase in homicide by firearm among all four age groups may be a result of easier access to firearms and increased life pressures induced by the worsening economy (21). During the first year of the COVID-19 pandemic, the sale of firearms increased dramatically in the United States, and many people lost jobs (8, 28, 29).

We saw inconsistent homicide and suicide mortality changes across sex and age groups. These changes may be anomalies in the data but may also reflect differences in life pressure shouldered by different genders and age groups. One encouraging finding was the slight decline of suicide rates among Americans aged 45–64 years old during the COVID-19 pandemic, a result that generally concords with reports from other high-income and upper-middle-income countries (30). It is difficult to interpret what might have led to that behavior pattern, however, especially given the simultaneous increase in suicide risk among younger adults in 2020.

One other factor must be considered as part of interpreting our results. The COVID-19 pandemic intensified pressure on the American health care system and forced hospitals to spend tremendous resources on prevention and treatment of COVID-19. These decisions reduced accessibility to healthcare and delayed emergency response to injured patients (31, 32). Simultaneously, people feared infecting COVID-19 in healthcare settings and delayed or avoided seeking treatment for serious but non-urgent conditions (33). Together, these patterns suggest patients with minor or moderate injuries may have chosen to stay at home and self-treat their injuries, creating a situation whereby many minor or moderate injuries that were captured by surveillance prior to the pandemic are not included in the database. Available data may therefore underestimate injury morbidity during the COVID-19 pandemic (34). Relatedly, delayed or inadequate medical treatment for injuries may have increased the mortality of patients with severe injuries (34).

### Policy implications

Our findings have three policy implications. First, the injury mortality and morbidity estimates underscore the importance of enhancing efforts for tailored injury prevention strategies that target high-risk populations exposed to specific injury causes in contextual environments like a pandemic. Priorities might include:

- *Preventing road traffic injury*: road and transport injury prevention planning that considers vulnerable road users such as motorcyclists, bicyclists and pedestrians, plus enhanced law enforcement to prohibit risky behavior such as not wearing a helmet; (35).
- *Preventing homicide/assault and suicide/self-harm*: making phone helpline and smartphone apps widely available and publicized for use; (36) altering stay-at-home orders for persons experiencing assault; (36) enabling reporting of assault *via* accessible strategies (e.g., *via* pharmacies or supermarkets) to prevent homicide/assault; (36) providing sufficient unemployment and social support; (37) and mitigating the adverse effect of COVID-19-related misinformation and irresponsible media reporting that leads to fear, anxiety, and depression.

Second, rational distribution of healthcare resources and accessibility is necessary to permit medical care capacity for health and injury situations beyond COVID-19. Emergency response time, prehospital transport times, and emergency rescue times should be shortened to reduce severe injuries, disability, or deaths due to delayed treatment (32, 34, 35). Citizens must feel they can obtain care for all medical conditions without fear of infection from COVID-19 during their care.

Third, because minor and moderate injury morbidity data may be skewed by hesitance or inability to seek treatment during the COVID-19 pandemic (7, 34), efforts should be made to identify ways to accurately capture data about injuries from the population in the community, both retrospectively during the pandemic and prospectively in case of future similar healthcare emergencies.

### Study limitations

This study was mainly limited by lack of sex- and age-specific data for relevant psychosocial and economic factors, such as economic development. We also lacked detailed data concerning the implementation of public health measures to control the COVID-19 pandemic. Without such data, we cannot eliminate their potential impact on our results and therefore cannot draw a direct causal conclusion between mortality/morbidity changes from 2018 to 2020 and the COVID-19 pandemic. Also, our findings were likely influenced by possible changes in data reporting practice, which previous research suggests could yield unexpected but substantial impact on injury mortality rates (38). During the COVID-19 pandemic, the reporting of mortality and morbidity was likely affected because medical staff were occupied with other priorities, such as clinical work (1, 39). Without full attention on data reporting, errors may have increased. In addition, we were limited to analysis of data from the United States because injury data from other countries are not freely available. The severity of the COVID-19 pandemic, patterns of infection and vaccination, and national responses to the pandemic differed widely across countries, so our findings might not generalize globally.

## Conclusions

The COVID-19 pandemic was related to increased overall injury mortality but decreased injury morbidity in the United States. Mortality and morbidity disparities between the two time periods (before and after the COVID-19 pandemic) were principally driven by distinct injury types and some discrepancies existed across sex and age groups. The results call for continued effort to tailor injury prevention strategies to particular sex- and age-groups as well as the environmental contexts that citizens face.

## Data availability statement

Publicly available datasets were analyzed in this study. This data can be found here: https://wonder.cdc.gov/; https://wisqars.cdc.gov/nonfatal-reports.

## Author contributions

GH, JH, and PN developed the ideas for this research. JH and PN analyzed data and drafted the initial manuscript. DS, YY, LL, PC, and ZR assisted in the interpretation of data and critically reviewed the manuscript. YY critically edited the statistical analysis strategies. GH, JH, PN, DS, and YY finalized the manuscript. All authors approved the final version of the manuscript.

## Funding

This work was supported by the Major Program of the National Social Science Foundation of China (No. 20&ZD120). The funding sources had no role in the study design, data collection, data analysis, data interpretation, in the writing of the report, or in the decision to submit for publication.

## Conflict of interest

The authors declare that the research was conducted in the absence of any commercial or financial relationships that could be construed as a potential conflict of interest.

## Publisher's note

All claims expressed in this article are solely those of the authors and do not necessarily represent those of their affiliated organizations, or those of the publisher, the editors and the reviewers. Any product that may be evaluated in this article, or claim that may be made by its manufacturer, is not guaranteed or endorsed by the publisher.
