# Supplementary material for: Injury mortality and morbidity changes due to the COVID-19 pandemic in the United States
Source: Front Public Health. 2022 Nov 2;10:1001567. doi: 10.3389/fpubh.2022.1001567 (PMC9666887; doi:10.3389/fpubh.2022.1001567)
Supplement: Supplementary file 1 [file Data_Sheet_1.docx]

Table of contents

**Supplementary Figure 1.** Sex-specific results for all Americans

**Supplementary Figure 2.** Sex-specific results for Americans aged 0-24 years old

**Supplementary Figure 3.** Sex-specific results for Americans aged 25-44 years old

**Supplementary Figure 4.** Sex-specific results Americans aged 45-64 years old

**Supplementary Figure 5.** Sex-specific results for Americans aged 65 years and older

**Supplementary Table 1.** RMtRR for all Americans

**Supplementary Table 2.** RMtRR for Americans aged 0-24 years old

**Supplementary Table 3.** RMtRR for Americans aged 25-44 years old

**Supplementary Table 4.** RMtRR for Americans aged 45-64 years old

**Supplementary Table 5.** RMtRR for Americans aged 65 years and older

**Supplementary Table 6.** RMbRR for all Americans

**Supplementary Table 7.** RMbRR for Americans aged 0-24 years old

**Supplementary Table 8.** RMbRR for Americans aged 25-44 years old

**Supplementary Table 9.** RMbRR for Americans aged 45-64 years old

**Supplementary Table 10.** RMbRR for Americans aged 65 years and older


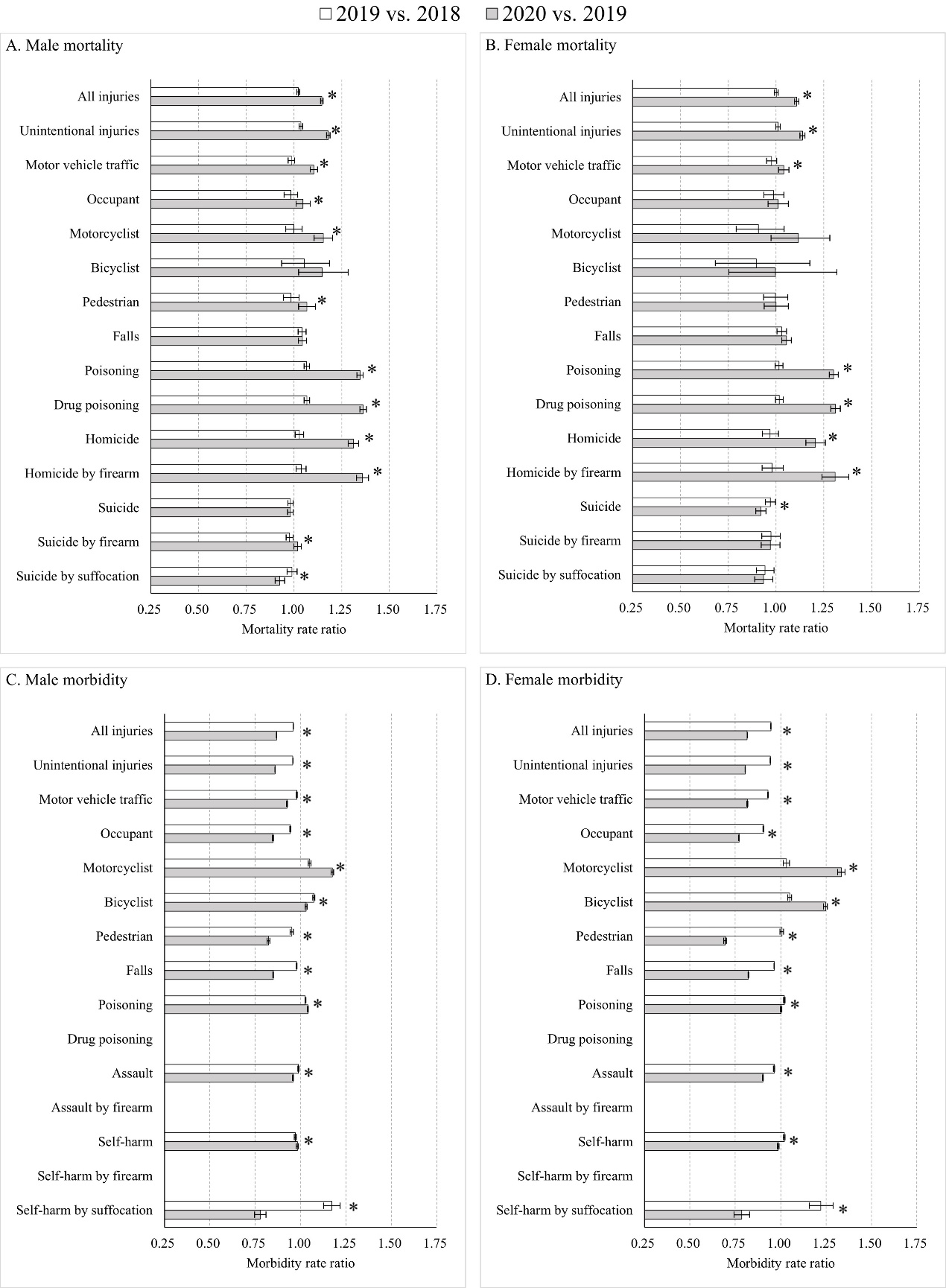


**Supplementary Figure 1.** Sex-specific age-standardized injury mortality/morbidity rate ratios among Americans for all age groups, 2019 vs. 2018 and 2020 vs. 2019. **(A)** Male mortality; **(B)** Female mortality; **(C)** Male morbidity; **(D)** Female morbidity.

Notes: Results were omitted for categories having unstable injury mortality or morbidity rates (due to 20 deaths/injuries or less, the national estimates less than 1,200, the coefficient of variation greater than 30%, or the tool not involving details regarding mechanism of relevant injury). “*” indicates that the test of “H_0_: RMtRR=1 or H_0_: RMbRR=1 (i.e., the null hypotheses of equal MtRRs or equal MbRRs between 2020 vs. 2019 and 2019 vs. 2018)” was statistically significant.


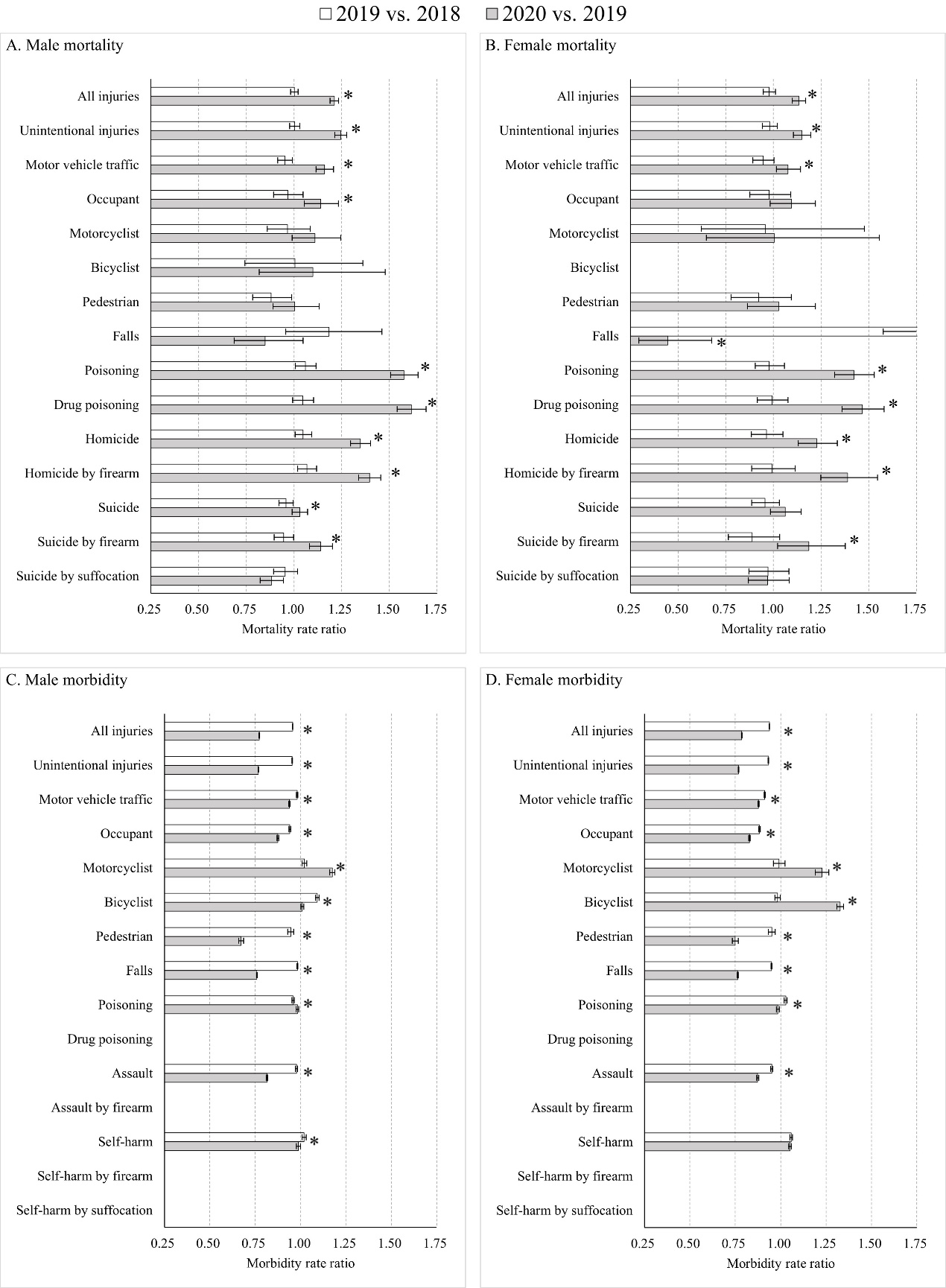


**Supplementary Figure 2.** Sex-specific age-standardized injury mortality/morbidity rate ratios among Americans aged 0-24 years old, 2019 vs. 2018 and 2020 vs. 2019. **(A)** Male mortality; **(B)** Female mortality; **(C)** Male morbidity; **(D)** Female morbidity.

Notes: Results were omitted for categories having unstable injury mortality or morbidity rates (due to 20 deaths/injuries or less, the national estimates less than 1,200, the coefficient of variation greater than 30%, or the tool not involving details regarding mechanism of relevant injury). “*” indicates that the test of “H_0_: RMtRR=1 or H_0_: RMbRR=1 (i.e., the null hypotheses of equal MtRRs or equal MbRRs between 2020 vs. 2019 and 2019 vs. 2018)” was statistically significant.


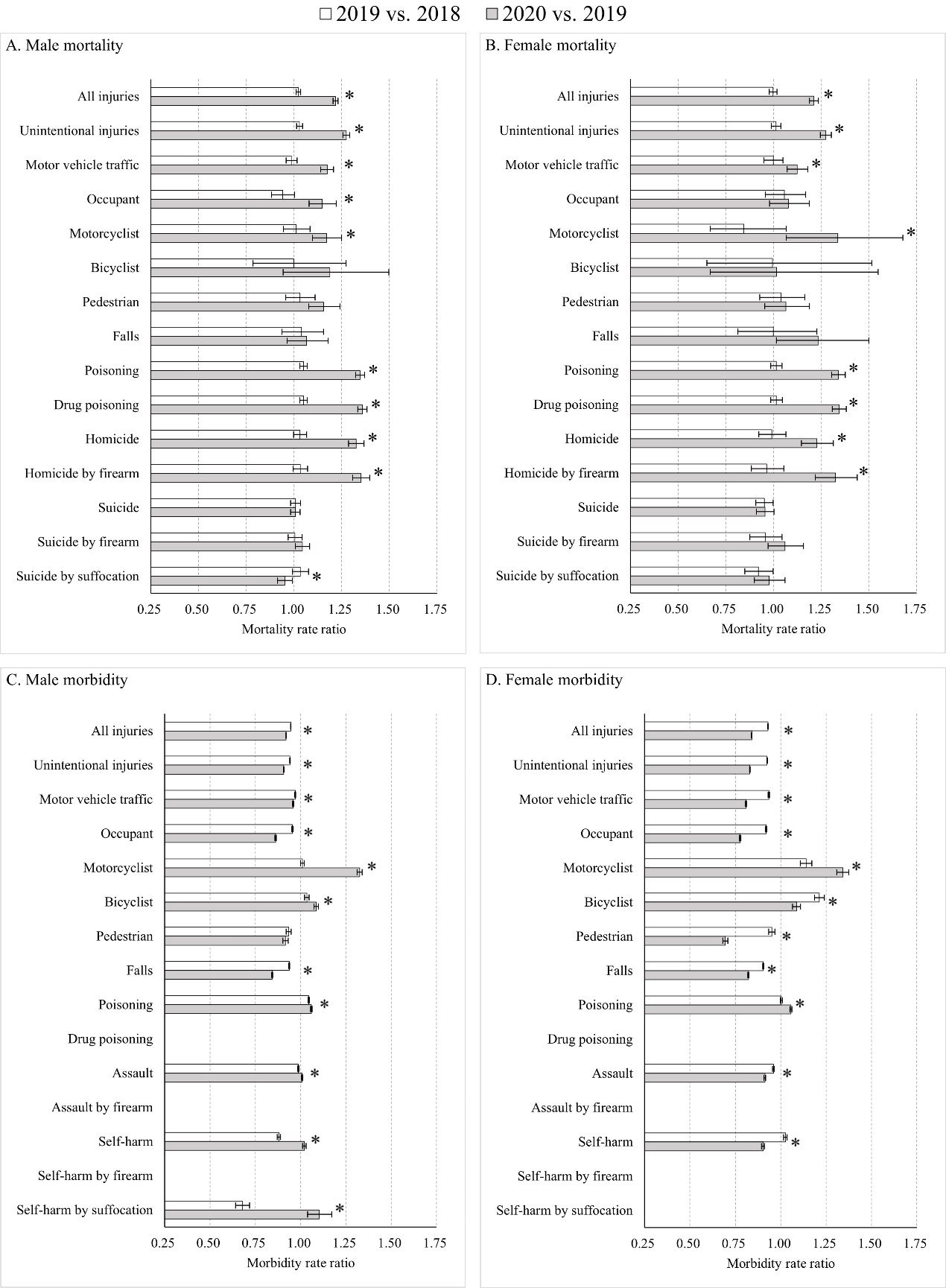


**Supplementary Figure 3.** Sex-specific age-standardized injury mortality/morbidity rate ratios among Americans aged 25-44 years old, 2019 vs. 2018 and 2020 vs. 2019. **(A)** Male mortality; **(B)** Female mortality; **(C)** Male morbidity; **(D)** Female morbidity.

Notes: Results were omitted for categories having unstable injury mortality or morbidity rates (due to 20 deaths/injuries or less, the national estimates less than 1,200, the coefficient of variation greater than 30%, or the tool not involving details regarding mechanism of relevant injury). “*” indicates that the test of “H_0_: RMtRR=1 or H_0_: RMbRR=1 (i.e., the null hypotheses of equal MtRRs or equal MbRRs between 2020 vs. 2019 and 2019 vs. 2018)” was statistically significant.


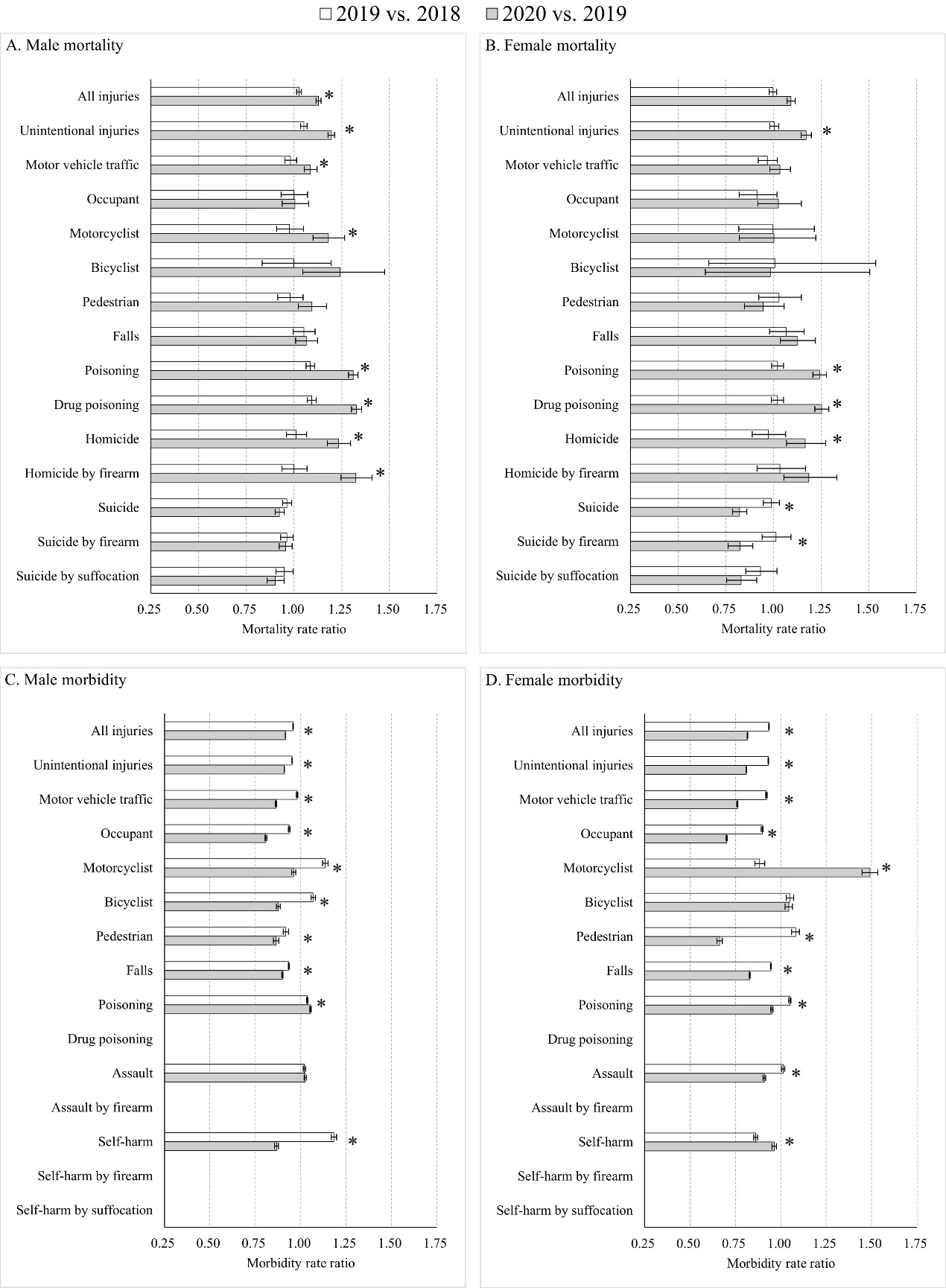


**Supplementary Figure 4.** Sex-specific age-standardized injury mortality/morbidity rate ratios among Americans aged 45-64 years old, 2019 vs. 2018 and 2020 vs. 2019. **(A)** Male mortality; **(B)** Female mortality; **(C)** Male morbidity; **(D)** Female morbidity.

Notes: Results were omitted for categories having unstable injury mortality or morbidity rates (due to 20 deaths/injuries or less, the national estimates less than 1,200, the coefficient of variation greater than 30%, or the tool not involving details regarding mechanism of relevant injury). “*” indicates that the test of “H_0_: RMtRR=1 or H_0_: RMbRR=1 (i.e., the null hypotheses of equal MtRRs or equal MbRRs between 2020 vs. 2019 and 2019 vs. 2018)” was statistically significant.


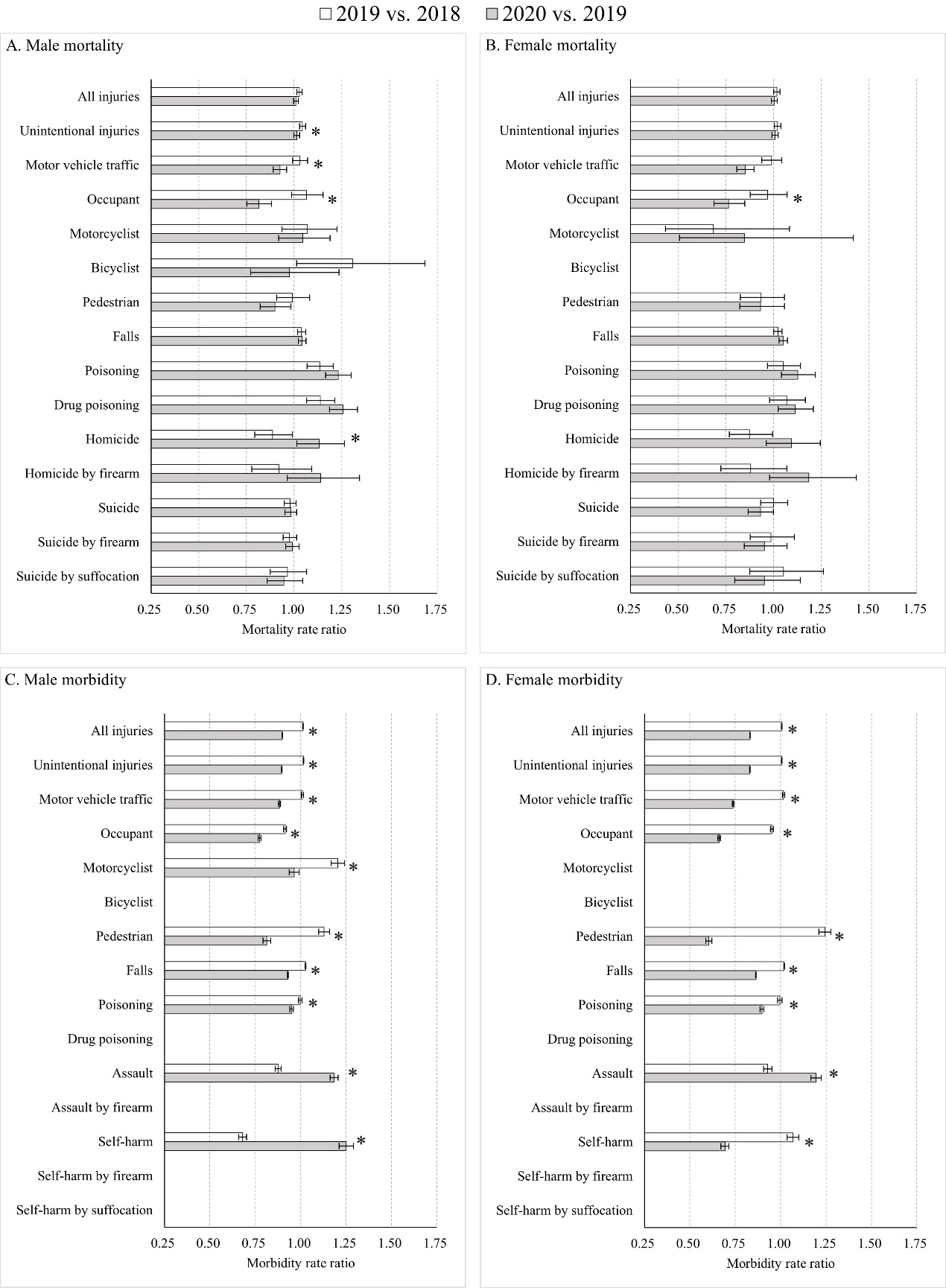


**Supplementary Figure 5.** Sex-specific age-standardized injury mortality/morbidity rate ratios among Americans aged 65 years and older, 2019 vs. 2018 and 2020 vs. 2019. **(A)** Male mortality; **(B)** Female mortality; **(C)** Male morbidity; **(D)** Female morbidity.

Notes: Results were omitted for categories having unstable injury mortality or morbidity rates (due to 20 deaths/injuries or less, the national estimates less than 1,200, the coefficient of variation greater than 30%, or the tool not involving details regarding mechanism of relevant injury). “*” indicates that the test of “H_0_: RMtRR=1 or H_0_: RMbRR=1 (i.e., the null hypotheses of equal MtRRs or equal MbRRs between 2020 vs. 2019 and 2019 vs. 2018)” was statistically significant.

**Supplementary Table 1.** Ratio of age-standardized injury mortality rate ratios (RMtRR) from 2018 to 2019 and from 2019 to 2020, all Americans.

| Cause of injury | Sex | From 2018 to 2019 | | |  | From 2019 to 2020 | | |  | 2019-2020 vs. 2018-2019 | | |
| --- | --- | --- | --- | --- | --- | --- | --- | --- | --- | --- | --- | --- |
|  |  | MtRR | 95% confidence interval | |  | MtRR | 95% confidence interval | |  | RMtRR | 95% confidence interval | |
| All injuries | Both sexes | 1.0160 | 1.0102 | 1.0219 |  | 1.1367 | 1.1304 | 1.1430 |  | 1.1187 | 1.1078 | 1.1298 |
|  | Male | 1.0226 | 1.0156 | 1.0296 |  | 1.1459 | 1.1384 | 1.1534 |  | 1.1206 | 1.1076 | 1.1338 |
|  | Female | 1.0010 | 0.9904 | 1.0117 |  | 1.1077 | 1.0963 | 1.1192 |  | 1.1066 | 1.0866 | 1.1270 |
| Unintentional injuries | Both sexes | 1.0270 | 1.0199 | 1.0342 |  | 1.1683 | 1.1605 | 1.1760 |  | 1.1375 | 1.1242 | 1.1511 |
|  | Male | 1.0365 | 1.0278 | 1.0453 |  | 1.1789 | 1.1695 | 1.1884 |  | 1.1374 | 1.1212 | 1.1538 |
|  | Female | 1.0103 | 0.9981 | 1.0227 |  | 1.1382 | 1.1249 | 1.1516 |  | 1.1266 | 1.1034 | 1.1503 |
| Motor vehicle traffic | Both sexes | 0.9849 | 0.9707 | 0.9993 |  | 1.0850 | 1.0697 | 1.1006 |  | 1.1017 | 1.0745 | 1.1295 |
|  | Male | 0.9865 | 0.9697 | 1.0036 |  | 1.1040 | 1.0856 | 1.1227 |  | 1.1191 | 1.0865 | 1.1527 |
|  | Female | 0.9768 | 0.9508 | 1.0035 |  | 1.0398 | 1.0123 | 1.0681 |  | 1.0646 | 1.0160 | 1.1155 |
| Occupant | Both sexes | 0.9703 | 0.9417 | 0.9998 |  | 1.0431 | 1.0125 | 1.0747 |  | 1.0751 | 1.0207 | 1.1323 |
|  | Male | 0.9831 | 0.9482 | 1.0193 |  | 1.0473 | 1.0105 | 1.0855 |  | 1.0654 | 1.0008 | 1.1341 |
|  | Female | 0.9866 | 0.9357 | 1.0403 |  | 1.0100 | 0.9580 | 1.0649 |  | 1.0238 | 0.9339 | 1.1222 |
| Motorcyclist | Both sexes | 0.9874 | 0.9473 | 1.0292 |  | 1.1753 | 1.1293 | 1.2232 |  | 1.1903 | 1.1087 | 1.2779 |
|  | Male | 0.9992 | 0.9566 | 1.0436 |  | 1.1531 | 1.1057 | 1.2025 |  | 1.1540 | 1.0712 | 1.2433 |
|  | Female | 0.9078 | 0.7912 | 1.0417 |  | 1.1168 | 0.9740 | 1.2806 |  | 1.2302 | 0.9680 | 1.5634 |
| Bicyclist | Both sexes | 1.0085 | 0.9070 | 1.1213 |  | 1.0557 | 0.9512 | 1.1717 |  | 1.0468 | 0.8722 | 1.2564 |
|  | Male | 1.0530 | 0.9352 | 1.1857 |  | 1.1473 | 1.0246 | 1.2847 |  | 1.0895 | 0.8907 | 1.3326 |
|  | Female | 0.8961 | 0.6822 | 1.1773 |  | 0.9961 | 0.7529 | 1.3180 |  | 1.1116 | 0.6872 | 1.7980 |
| Pedestrian | Both sexes | 1.0008 | 0.9667 | 1.0361 |  | 1.0301 | 0.9953 | 1.0661 |  | 1.0293 | 0.9695 | 1.0928 |
|  | Male | 0.9848 | 0.9449 | 1.0264 |  | 1.0669 | 1.0243 | 1.1114 |  | 1.0834 | 1.0087 | 1.1636 |
|  | Female | 0.9962 | 0.9352 | 1.0612 |  | 0.9992 | 0.9381 | 1.0644 |  | 1.0030 | 0.8991 | 1.1191 |
| Falls | Both sexes | 1.0365 | 1.0205 | 1.0529 |  | 1.0511 | 1.0352 | 1.0673 |  | 1.0141 | 0.9873 | 1.0416 |
|  | Male | 1.0423 | 1.0215 | 1.0635 |  | 1.0438 | 1.0234 | 1.0645 |  | 1.0015 | 0.9675 | 1.0366 |
|  | Female | 1.0289 | 1.0043 | 1.0541 |  | 1.0540 | 1.0294 | 1.0793 |  | 1.0244 | 0.9828 | 1.0678 |
| Poisoning | Both sexes | 1.0506 | 1.0392 | 1.0621 |  | 1.3324 | 1.3191 | 1.3459 |  | 1.2683 | 1.2453 | 1.2918 |
|  | Male | 1.0661 | 1.0522 | 1.0802 |  | 1.3456 | 1.3296 | 1.3619 |  | 1.2622 | 1.2348 | 1.2902 |
|  | Female | 1.0159 | 0.9960 | 1.0361 |  | 1.3012 | 1.2774 | 1.3255 |  | 1.2809 | 1.2387 | 1.3245 |
| Drug poisoning | Both sexes | 1.0511 | 1.0393 | 1.0629 |  | 1.3486 | 1.3347 | 1.3625 |  | 1.2830 | 1.2591 | 1.3074 |
|  | Male | 1.0674 | 1.0531 | 1.0819 |  | 1.3623 | 1.3456 | 1.3793 |  | 1.2763 | 1.2478 | 1.3055 |
|  | Female | 1.0175 | 0.9971 | 1.0383 |  | 1.3109 | 1.2863 | 1.3358 |  | 1.2883 | 1.2450 | 1.3332 |
| Homicide | Both sexes | 1.0177 | 0.9977 | 1.0380 |  | 1.2891 | 1.2654 | 1.3132 |  | 1.2667 | 1.2249 | 1.3099 |
|  | Male | 1.0278 | 1.0051 | 1.0511 |  | 1.3117 | 1.2846 | 1.3393 |  | 1.2762 | 1.2288 | 1.3254 |
|  | Female | 0.9692 | 0.9279 | 1.0124 |  | 1.2053 | 1.1558 | 1.2570 |  | 1.2436 | 1.1540 | 1.3402 |
| Homicide by firearm | Both sexes | 1.0337 | 1.0104 | 1.0575 |  | 1.3476 | 1.3196 | 1.3763 |  | 1.3037 | 1.2546 | 1.3547 |
|  | Male | 1.0377 | 1.0119 | 1.0641 |  | 1.3591 | 1.3280 | 1.3910 |  | 1.3097 | 1.2555 | 1.3664 |
|  | Female | 0.9802 | 0.9261 | 1.0374 |  | 1.3086 | 1.2405 | 1.3804 |  | 1.3350 | 1.2122 | 1.4703 |
| Suicide | Both sexes | 0.9784 | 0.9659 | 0.9911 |  | 0.9700 | 0.9575 | 0.9828 |  | 0.9914 | 0.9694 | 1.0140 |
|  | Male | 0.9817 | 0.9676 | 0.9961 |  | 0.9815 | 0.9672 | 0.9960 |  | 0.9997 | 0.9748 | 1.0253 |
|  | Female | 0.9706 | 0.9442 | 0.9977 |  | 0.9205 | 0.8948 | 0.9470 |  | 0.9484 | 0.9037 | 0.9953 |
| Suicide by firearm | Both sexes | 0.9721 | 0.9544 | 0.9901 |  | 1.0165 | 0.9980 | 1.0354 |  | 1.0457 | 1.0128 | 1.0796 |
|  | Male | 0.9772 | 0.9583 | 0.9965 |  | 1.0185 | 0.9987 | 1.0386 |  | 1.0422 | 1.0074 | 1.0782 |
|  | Female | 0.9730 | 0.9258 | 1.0226 |  | 0.9705 | 0.9229 | 1.0206 |  | 0.9975 | 0.9147 | 1.0877 |
| Suicide by suffocation | Both sexes | 0.9784 | 0.9557 | 1.0017 |  | 0.9259 | 0.9039 | 0.9485 |  | 0.9463 | 0.9082 | 0.9861 |
|  | Male | 0.9900 | 0.9638 | 1.0169 |  | 0.9253 | 0.9003 | 0.9509 |  | 0.9346 | 0.8918 | 0.9794 |
|  | Female | 0.9422 | 0.8967 | 0.9899 |  | 0.9336 | 0.8872 | 0.9824 |  | 0.9909 | 0.9084 | 1.0809 |

Notes: MtRR= mortality rate ratio, RMtRR=ratio of mortality rate ratio.

**Supplementary Table 2.** Ratio of age-standardized injury mortality rate ratios (RMtRR) from 2018 to 2019 and from 2019 to 2020, Americans aged 0-24 years old.

| Cause of injury | Sex | From 2018 to 2019 | | |  | From 2019 to 2020 | | |  | 2019-2020 vs. 2018-2019 | | |
| --- | --- | --- | --- | --- | --- | --- | --- | --- | --- | --- | --- | --- |
|  |  | MtRR | 95% confidence interval | |  | MtRR | 95% confidence interval | |  | RMtRR | 95% confidence interval | |
| All injuries | Both sexes | 0.9950 | 0.9786 | 1.0116 |  | 1.1888 | 1.1700 | 1.2080 |  | 1.1948 | 1.1613 | 1.2293 |
|  | Male | 1.0019 | 0.9827 | 1.0214 |  | 1.2114 | 1.1892 | 1.2341 |  | 1.2092 | 1.1699 | 1.2499 |
|  | Female | 0.9779 | 0.9468 | 1.0101 |  | 1.1331 | 1.0979 | 1.1695 |  | 1.1587 | 1.0959 | 1.2251 |
| Unintentional injuries | Both sexes | 0.9935 | 0.9715 | 1.0160 |  | 1.2159 | 1.1900 | 1.2422 |  | 1.2238 | 1.1778 | 1.2716 |
|  | Male | 1.0040 | 0.9773 | 1.0315 |  | 1.2448 | 1.2133 | 1.2772 |  | 1.2398 | 1.1841 | 1.2982 |
|  | Female | 0.9798 | 0.9412 | 1.0200 |  | 1.1492 | 1.1051 | 1.1951 |  | 1.1729 | 1.0945 | 1.2569 |
| Motor vehicle traffic | Both sexes | 0.9529 | 0.9217 | 0.9852 |  | 1.1272 | 1.0907 | 1.1648 |  | 1.1829 | 1.1165 | 1.2532 |
|  | Male | 0.9527 | 0.9149 | 0.9922 |  | 1.1606 | 1.1155 | 1.2076 |  | 1.2182 | 1.1358 | 1.3065 |
|  | Female | 0.9461 | 0.8920 | 1.0035 |  | 1.0763 | 1.0149 | 1.1414 |  | 1.1376 | 1.0269 | 1.2603 |
| Occupant | Both sexes | 0.9547 | 0.8949 | 1.0184 |  | 1.1171 | 1.0481 | 1.1907 |  | 1.1701 | 1.0462 | 1.3087 |
|  | Male | 0.9679 | 0.8938 | 1.0481 |  | 1.1404 | 1.0549 | 1.2328 |  | 1.1783 | 1.0270 | 1.3518 |
|  | Female | 0.9773 | 0.8754 | 1.0910 |  | 1.0951 | 0.9825 | 1.2206 |  | 1.1205 | 0.9264 | 1.3553 |
| Motorcyclist | Both sexes | 0.8747 | 0.7816 | 0.9789 |  | 1.2182 | 1.0901 | 1.3615 |  | 1.3928 | 1.1444 | 1.6951 |
|  | Male | 0.9664 | 0.8606 | 1.0853 |  | 1.1102 | 0.9903 | 1.2446 |  | 1.1488 | 0.9400 | 1.4039 |
|  | Female | 0.9576 | 0.6210 | 1.4765 |  | 1.0036 | 0.6475 | 1.5555 |  | 1.0480 | 0.4920 | 2.2322 |
| Bicyclist | Both sexes | 1.0058 | 0.7842 | 1.2901 |  | 0.9959 | 0.7760 | 1.2780 |  | 0.9901 | 0.6432 | 1.5242 |
|  | Male | 1.0061 | 0.7435 | 1.3614 |  | 1.1000 | 0.8184 | 1.4786 |  | 1.0933 | 0.6500 | 1.8391 |
|  | Female | — | — | — |  | — | — | — |  | — | — | — |
| Pedestrian | Both sexes | 0.9503 | 0.8639 | 1.0454 |  | 0.9686 | 0.8785 | 1.0678 |  | 1.0192 | 0.8623 | 1.2047 |
|  | Male | 0.8804 | 0.7837 | 0.9890 |  | 1.0044 | 0.8907 | 1.1326 |  | 1.1409 | 0.9285 | 1.4018 |
|  | Female | 0.9223 | 0.7781 | 1.0931 |  | 1.0272 | 0.8644 | 1.2207 |  | 1.1138 | 0.8267 | 1.5004 |
| Falls | Both sexes | 1.2586 | 1.0363 | 1.5286 |  | 0.9996 | 0.8321 | 1.2008 |  | 0.7942 | 0.5744 | 1.0980 |
|  | Male | 1.1833 | 0.9578 | 1.4619 |  | 0.8486 | 0.6867 | 1.0488 |  | 0.7172 | 0.4996 | 1.0294 |
|  | Female | 2.4131 | 1.5762 | 3.6944 |  | 0.4460 | 0.2941 | 0.6764 |  | 0.1848 | 0.0937 | 0.3646 |
| Poisoning | Both sexes | 1.0357 | 0.9921 | 1.0812 |  | 1.5285 | 1.4704 | 1.5890 |  | 1.4759 | 1.3734 | 1.5859 |
|  | Male | 1.0597 | 1.0068 | 1.1154 |  | 1.5766 | 1.5061 | 1.6503 |  | 1.4878 | 1.3662 | 1.6201 |
|  | Female | 0.9781 | 0.9038 | 1.0585 |  | 1.4210 | 1.3203 | 1.5294 |  | 1.4529 | 1.2706 | 1.6613 |
| Drug poisoning | Both sexes | 1.0106 | 0.9670 | 1.0562 |  | 1.5892 | 1.5272 | 1.6537 |  | 1.5724 | 1.4603 | 1.6931 |
|  | Male | 1.0461 | 0.9927 | 1.1024 |  | 1.6153 | 1.5415 | 1.6926 |  | 1.5441 | 1.4149 | 1.6850 |
|  | Female | 0.9924 | 0.9146 | 1.0769 |  | 1.4659 | 1.3597 | 1.5804 |  | 1.4770 | 1.2867 | 1.6956 |
| Homicide | Both sexes | 1.0342 | 0.9959 | 1.0741 |  | 1.3237 | 1.2779 | 1.3711 |  | 1.2798 | 1.2007 | 1.3642 |
|  | Male | 1.0484 | 1.0052 | 1.0934 |  | 1.3476 | 1.2963 | 1.4010 |  | 1.2854 | 1.1976 | 1.3797 |
|  | Female | 0.9632 | 0.8833 | 1.0503 |  | 1.2277 | 1.1294 | 1.3345 |  | 1.2746 | 1.0985 | 1.4789 |
| Homicide by firearm | Both sexes | 1.0594 | 1.0155 | 1.1052 |  | 1.3857 | 1.3330 | 1.4404 |  | 1.3080 | 1.2183 | 1.4042 |
|  | Male | 1.0682 | 1.0205 | 1.1183 |  | 1.3960 | 1.3389 | 1.4556 |  | 1.3068 | 1.2104 | 1.4109 |
|  | Female | 0.9935 | 0.8859 | 1.1142 |  | 1.3887 | 1.2482 | 1.5452 |  | 1.3979 | 1.1510 | 1.6977 |
| Suicide | Both sexes | 0.9496 | 0.9172 | 0.9832 |  | 1.0334 | 0.9978 | 1.0702 |  | 1.0882 | 1.0242 | 1.1562 |
|  | Male | 0.9588 | 0.9220 | 0.9970 |  | 1.0302 | 0.9905 | 1.0715 |  | 1.0745 | 1.0037 | 1.1503 |
|  | Female | 0.9561 | 0.8861 | 1.0316 |  | 1.0615 | 0.9840 | 1.1452 |  | 1.1103 | 0.9729 | 1.2671 |
| Suicide by firearm | Both sexes | 0.9327 | 0.8863 | 0.9815 |  | 1.1434 | 1.0873 | 1.2025 |  | 1.2260 | 1.1221 | 1.3395 |
|  | Male | 0.9456 | 0.8957 | 0.9983 |  | 1.1396 | 1.0803 | 1.2021 |  | 1.2051 | 1.0970 | 1.3238 |
|  | Female | 0.8885 | 0.7642 | 1.0329 |  | 1.1860 | 1.0217 | 1.3767 |  | 1.3349 | 1.0265 | 1.7359 |
| Suicide by suffocation | Both sexes | 0.9504 | 0.8984 | 1.0053 |  | 0.9144 | 0.8626 | 0.9694 |  | 0.9622 | 0.8715 | 1.0624 |
|  | Male | 0.9537 | 0.8933 | 1.0182 |  | 0.8820 | 0.8236 | 0.9446 |  | 0.9248 | 0.8238 | 1.0382 |
|  | Female | 0.9706 | 0.8708 | 1.0819 |  | 0.9691 | 0.8678 | 1.0823 |  | 0.9984 | 0.8260 | 1.2069 |

Notes: MtRR= mortality rate ratio, RMtRR=ratio of mortality rate ratio.

—: Results were omitted for categories having unstable injury mortality rates (with 20 deaths or less).

**Supplementary Table 3.** Ratio of age-standardized injury mortality rate ratios (RMtRR) from 2018 to 2019 and from 2019 to 2020, Americans aged 25-44 years old.

| Cause of injury | Sex | From 2018 to 2019 | | |  | From 2019 to 2020 | | |  | 2019-2020 vs. 2018-2019 | | |
| --- | --- | --- | --- | --- | --- | --- | --- | --- | --- | --- | --- | --- |
|  |  | MtRR | 95% confidence interval | |  | MtRR | 95% confidence interval | |  | RMtRR | 95% confidence interval | |
| All injuries | Both sexes | 1.0171 | 1.0069 | 1.0275 |  | 1.2171 | 1.2054 | 1.2288 |  | 1.1965 | 1.1761 | 1.2173 |
|  | Male | 1.0240 | 1.0120 | 1.0360 |  | 1.2175 | 1.2041 | 1.2310 |  | 1.1890 | 1.1656 | 1.2128 |
|  | Female | 0.9977 | 0.9776 | 1.0181 |  | 1.2119 | 1.1887 | 1.2356 |  | 1.2148 | 1.1735 | 1.2575 |
| Unintentional injuries | Both sexes | 1.0249 | 1.0120 | 1.0379 |  | 1.2744 | 1.2594 | 1.2895 |  | 1.2434 | 1.2171 | 1.2703 |
|  | Male | 1.0293 | 1.0142 | 1.0447 |  | 1.2745 | 1.2570 | 1.2923 |  | 1.2382 | 1.2076 | 1.2696 |
|  | Female | 1.0136 | 0.9894 | 1.0385 |  | 1.2741 | 1.2455 | 1.3033 |  | 1.2569 | 1.2064 | 1.3095 |
| Motor vehicle traffic | Both sexes | 0.9920 | 0.9670 | 1.0176 |  | 1.1600 | 1.1318 | 1.1889 |  | 1.1694 | 1.1195 | 1.2216 |
|  | Male | 0.9872 | 0.9583 | 1.0170 |  | 1.1739 | 1.1408 | 1.2079 |  | 1.1891 | 1.1301 | 1.2511 |
|  | Female | 0.9989 | 0.9504 | 1.0500 |  | 1.1246 | 1.0716 | 1.1803 |  | 1.1259 | 1.0337 | 1.2263 |
| Occupant | Both sexes | 0.9591 | 0.9089 | 1.0121 |  | 1.1433 | 1.0849 | 1.2048 |  | 1.1921 | 1.0866 | 1.3078 |
|  | Male | 0.9405 | 0.8825 | 1.0024 |  | 1.1483 | 1.0789 | 1.2221 |  | 1.2209 | 1.0937 | 1.3628 |
|  | Female | 1.0577 | 0.9571 | 1.1689 |  | 1.0790 | 0.9798 | 1.1882 |  | 1.0201 | 0.8605 | 1.2094 |
| Motorcyclist | Both sexes | 1.0228 | 0.9572 | 1.0929 |  | 1.1928 | 1.1200 | 1.2703 |  | 1.1661 | 1.0420 | 1.3051 |
|  | Male | 1.0124 | 0.9451 | 1.0845 |  | 1.1711 | 1.0965 | 1.2508 |  | 1.1568 | 1.0287 | 1.3007 |
|  | Female | 0.8444 | 0.6683 | 1.0668 |  | 1.3379 | 1.0663 | 1.6788 |  | 1.5845 | 1.0553 | 2.3792 |
| Bicyclist | Both sexes | 0.9986 | 0.8096 | 1.2317 |  | 0.9988 | 0.8102 | 1.2313 |  | 1.0002 | 0.6958 | 1.4378 |
|  | Male | 0.9996 | 0.7849 | 1.2729 |  | 1.1879 | 0.9427 | 1.4967 |  | 1.1884 | 0.7868 | 1.7948 |
|  | Female | 0.9938 | 0.6512 | 1.5166 |  | 1.0170 | 0.6680 | 1.5483 |  | 1.0234 | 0.4928 | 2.1252 |
| Pedestrian | Both sexes | 1.0445 | 0.9819 | 1.1111 |  | 1.1077 | 1.0438 | 1.1755 |  | 1.0605 | 0.9546 | 1.1782 |
|  | Male | 1.0315 | 0.9581 | 1.1105 |  | 1.1561 | 1.0774 | 1.2405 |  | 1.1208 | 0.9885 | 1.2707 |
|  | Female | 1.0396 | 0.9282 | 1.1644 |  | 1.0653 | 0.9542 | 1.1894 |  | 1.0247 | 0.8443 | 1.2438 |
| Falls | Both sexes | 1.0097 | 0.9202 | 1.1079 |  | 1.1498 | 1.0517 | 1.2571 |  | 1.1388 | 0.9719 | 1.3343 |
|  | Male | 1.0395 | 0.9365 | 1.1539 |  | 1.0666 | 0.9638 | 1.1804 |  | 1.0261 | 0.8584 | 1.2264 |
|  | Female | 0.9993 | 0.8134 | 1.2275 |  | 1.2342 | 1.0153 | 1.5002 |  | 1.2351 | 0.8702 | 1.7530 |
| Poisoning | Both sexes | 1.0397 | 1.0235 | 1.0561 |  | 1.3453 | 1.3260 | 1.3649 |  | 1.2940 | 1.2603 | 1.3286 |
|  | Male | 1.0500 | 1.0307 | 1.0697 |  | 1.3468 | 1.3240 | 1.3700 |  | 1.2827 | 1.2433 | 1.3233 |
|  | Female | 1.0146 | 0.9853 | 1.0448 |  | 1.3401 | 1.3042 | 1.3771 |  | 1.3208 | 1.2570 | 1.3878 |
| Drug poisoning | Both sexes | 1.0414 | 1.0249 | 1.0582 |  | 1.3542 | 1.3344 | 1.3743 |  | 1.3003 | 1.2658 | 1.3358 |
|  | Male | 1.0504 | 1.0306 | 1.0705 |  | 1.3586 | 1.3351 | 1.3824 |  | 1.2934 | 1.2529 | 1.3353 |
|  | Female | 1.0151 | 0.9853 | 1.0459 |  | 1.3445 | 1.3078 | 1.3822 |  | 1.3244 | 1.2594 | 1.3928 |
| Homicide | Both sexes | 1.0218 | 0.9915 | 1.0531 |  | 1.3089 | 1.2727 | 1.3462 |  | 1.2810 | 1.2174 | 1.3479 |
|  | Male | 1.0315 | 0.9978 | 1.0664 |  | 1.3257 | 1.2856 | 1.3672 |  | 1.2852 | 1.2152 | 1.3592 |
|  | Female | 0.9918 | 0.9232 | 1.0655 |  | 1.2275 | 1.1466 | 1.3142 |  | 1.2377 | 1.0953 | 1.3987 |
| Homicide by firearm | Both sexes | 1.0319 | 0.9978 | 1.0671 |  | 1.3433 | 1.3023 | 1.3856 |  | 1.3018 | 1.2303 | 1.3774 |
|  | Male | 1.0328 | 0.9960 | 1.0710 |  | 1.3518 | 1.3073 | 1.3978 |  | 1.3088 | 1.2312 | 1.3913 |
|  | Female | 0.9650 | 0.8835 | 1.0540 |  | 1.3252 | 1.2194 | 1.4401 |  | 1.3733 | 1.1816 | 1.5961 |
| Suicide | Both sexes | 0.9945 | 0.9726 | 1.0168 |  | 1.0015 | 0.9796 | 1.0239 |  | 1.0071 | 0.9692 | 1.0465 |
|  | Male | 1.0078 | 0.9829 | 1.0334 |  | 1.0072 | 0.9824 | 1.0325 |  | 0.9993 | 0.9571 | 1.0434 |
|  | Female | 0.9518 | 0.9073 | 0.9984 |  | 0.9555 | 0.9099 | 1.0033 |  | 1.0039 | 0.9233 | 1.0916 |
| Suicide by firearm | Both sexes | 0.9988 | 0.9655 | 1.0333 |  | 1.0464 | 1.0121 | 1.0820 |  | 1.0477 | 0.9882 | 1.1107 |
|  | Male | 1.0057 | 0.9694 | 1.0434 |  | 1.0448 | 1.0077 | 1.0833 |  | 1.0389 | 0.9751 | 1.1068 |
|  | Female | 0.9568 | 0.8766 | 1.0444 |  | 1.0606 | 0.9722 | 1.1570 |  | 1.1085 | 0.9524 | 1.2903 |
| Suicide by suffocation | Both sexes | 1.0164 | 0.9802 | 1.0540 |  | 0.9461 | 0.9122 | 0.9812 |  | 0.9308 | 0.8741 | 0.9911 |
|  | Male | 1.0334 | 0.9921 | 1.0764 |  | 0.9516 | 0.9136 | 0.9912 |  | 0.9208 | 0.8583 | 0.9880 |
|  | Female | 0.9211 | 0.8502 | 0.9979 |  | 0.9766 | 0.8998 | 1.0601 |  | 1.0603 | 0.9211 | 1.2206 |

Notes: MtRR= mortality rate ratio, RMtRR=ratio of mortality rate ratio.

**Supplementary Table 4.** Ratio of age-standardized injury mortality rate ratios (RMtRR) from 2018 to 2019 and from 2019 to 2020, Americans aged 45-64 years old.

| Cause of injury | Sex | From 2018 to 2019 | | |  | From 2019 to 2020 | | |  | 2019-2020 vs. 2018-2019 | | |
| --- | --- | --- | --- | --- | --- | --- | --- | --- | --- | --- | --- | --- |
|  |  | MtRR | 95% confidence interval | |  | MtRR | 95% confidence interval | |  | RMtRR | 95% confidence interval | |
| All injuries | Both sexes | 1.0187 | 1.0081 | 1.0294 |  | 1.1181 | 1.1068 | 1.1295 |  | 1.0976 | 1.0780 | 1.1175 |
|  | Male | 1.0263 | 1.0137 | 1.0391 |  | 1.1286 | 1.1152 | 1.1422 |  | 1.0997 | 1.0766 | 1.1232 |
|  | Female | 0.9975 | 0.9782 | 1.0172 |  | 1.0922 | 1.0714 | 1.1134 |  | 1.0949 | 1.0586 | 1.1325 |
| Unintentional injuries | Both sexes | 1.0369 | 1.0238 | 1.0502 |  | 1.1896 | 1.1752 | 1.2041 |  | 1.1472 | 1.1226 | 1.1723 |
|  | Male | 1.0519 | 1.0361 | 1.0680 |  | 1.1955 | 1.1784 | 1.2128 |  | 1.1365 | 1.1075 | 1.1661 |
|  | Female | 1.0042 | 0.9811 | 1.0280 |  | 1.1721 | 1.1460 | 1.1988 |  | 1.1671 | 1.1213 | 1.2148 |
| Motor vehicle traffic | Both sexes | 0.9830 | 0.9571 | 1.0096 |  | 1.0673 | 1.0394 | 1.0959 |  | 1.0858 | 1.0368 | 1.1372 |
|  | Male | 0.9821 | 0.9520 | 1.0132 |  | 1.0868 | 1.0538 | 1.1207 |  | 1.1066 | 1.0485 | 1.1678 |
|  | Female | 0.9685 | 0.9194 | 1.0201 |  | 1.0333 | 0.9809 | 1.0886 |  | 1.0670 | 0.9748 | 1.1679 |
| Occupant | Both sexes | 0.9636 | 0.9089 | 1.0216 |  | 1.0232 | 0.9647 | 1.0852 |  | 1.0618 | 0.9590 | 1.1757 |
|  | Male | 0.9997 | 0.9327 | 1.0715 |  | 1.0057 | 0.9382 | 1.0781 |  | 1.0060 | 0.8921 | 1.1345 |
|  | Female | 0.9135 | 0.8196 | 1.0181 |  | 1.0264 | 0.9190 | 1.1463 |  | 1.1236 | 0.9287 | 1.3595 |
| Motorcyclist | Both sexes | 0.9892 | 0.9245 | 1.0583 |  | 1.1608 | 1.0871 | 1.2395 |  | 1.1735 | 1.0449 | 1.3180 |
|  | Male | 0.9771 | 0.9091 | 1.0501 |  | 1.1800 | 1.1003 | 1.2654 |  | 1.2077 | 1.0668 | 1.3671 |
|  | Female | 0.9971 | 0.8184 | 1.2149 |  | 1.0019 | 0.8217 | 1.2215 |  | 1.0047 | 0.7132 | 1.4155 |
| Bicyclist | Both sexes | 0.9999 | 0.8483 | 1.1787 |  | 1.1134 | 0.9481 | 1.3076 |  | 1.1135 | 0.8391 | 1.4776 |
|  | Male | 0.9981 | 0.8333 | 1.1955 |  | 1.2419 | 1.0456 | 1.4750 |  | 1.2442 | 0.9144 | 1.6930 |
|  | Female | 1.0073 | 0.6601 | 1.5372 |  | 0.9836 | 0.6429 | 1.5048 |  | 0.9764 | 0.4689 | 2.0335 |
| Pedestrian | Both sexes | 0.9925 | 0.9373 | 1.0509 |  | 1.0356 | 0.9782 | 1.0964 |  | 1.0435 | 0.9450 | 1.1522 |
|  | Male | 0.9794 | 0.9153 | 1.0480 |  | 1.0939 | 1.0233 | 1.1694 |  | 1.1169 | 0.9936 | 1.2554 |
|  | Female | 1.0289 | 0.9236 | 1.1463 |  | 0.9465 | 0.8487 | 1.0556 |  | 0.9199 | 0.7627 | 1.1094 |
| Falls | Both sexes | 1.0808 | 1.0323 | 1.1316 |  | 1.0701 | 1.0236 | 1.1188 |  | 0.9901 | 0.9156 | 1.0707 |
|  | Male | 1.0520 | 0.9962 | 1.1109 |  | 1.0642 | 1.0091 | 1.1223 |  | 1.0116 | 0.9215 | 1.1105 |
|  | Female | 1.0664 | 0.9791 | 1.1615 |  | 1.1251 | 1.0365 | 1.2212 |  | 1.0550 | 0.9123 | 1.2201 |
| Poisoning | Both sexes | 1.0654 | 1.0471 | 1.0839 |  | 1.2883 | 1.2678 | 1.3092 |  | 1.2093 | 1.1747 | 1.2449 |
|  | Male | 1.0855 | 1.0630 | 1.1085 |  | 1.3108 | 1.2857 | 1.3364 |  | 1.2075 | 1.1658 | 1.2507 |
|  | Female | 1.0217 | 0.9911 | 1.0532 |  | 1.2424 | 1.2071 | 1.2786 |  | 1.2160 | 1.1548 | 1.2804 |
| Drug poisoning | Both sexes | 1.0686 | 1.0497 | 1.0878 |  | 1.3058 | 1.2844 | 1.3276 |  | 1.2220 | 1.1859 | 1.2592 |
|  | Male | 1.0929 | 1.0694 | 1.1170 |  | 1.3281 | 1.3018 | 1.3548 |  | 1.2151 | 1.1717 | 1.2602 |
|  | Female | 1.0208 | 0.9895 | 1.0531 |  | 1.2534 | 1.2170 | 1.2910 |  | 1.2279 | 1.1646 | 1.2947 |
| Homicide | Both sexes | 1.0040 | 0.9596 | 1.0505 |  | 1.2107 | 1.1593 | 1.2643 |  | 1.2058 | 1.1160 | 1.3028 |
|  | Male | 1.0117 | 0.9599 | 1.0662 |  | 1.2342 | 1.1741 | 1.2974 |  | 1.2200 | 1.1156 | 1.3341 |
|  | Female | 0.9725 | 0.8884 | 1.0646 |  | 1.1663 | 1.0680 | 1.2737 |  | 1.1992 | 1.0263 | 1.4013 |
| Homicide by firearm | Both sexes | 1.0000 | 0.9438 | 1.0595 |  | 1.3002 | 1.2312 | 1.3731 |  | 1.3002 | 1.1785 | 1.4346 |
|  | Male | 0.9999 | 0.9366 | 1.0675 |  | 1.3249 | 1.2459 | 1.4089 |  | 1.3251 | 1.1856 | 1.4809 |
|  | Female | 1.0335 | 0.9137 | 1.1690 |  | 1.1856 | 1.0540 | 1.3337 |  | 1.1472 | 0.9301 | 1.4149 |
| Suicide | Both sexes | 0.9722 | 0.9515 | 0.9934 |  | 0.8999 | 0.8800 | 0.9203 |  | 0.9256 | 0.8912 | 0.9614 |
|  | Male | 0.9636 | 0.9398 | 0.9879 |  | 0.9245 | 0.9009 | 0.9486 |  | 0.9594 | 0.9182 | 1.0024 |
|  | Female | 0.9884 | 0.9471 | 1.0315 |  | 0.8224 | 0.7861 | 0.8604 |  | 0.8320 | 0.7715 | 0.8973 |
| Suicide by firearm | Both sexes | 0.9607 | 0.9312 | 0.9911 |  | 0.9465 | 0.9166 | 0.9773 |  | 0.9852 | 0.9327 | 1.0406 |
|  | Male | 0.9633 | 0.9309 | 0.9969 |  | 0.9563 | 0.9233 | 0.9904 |  | 0.9927 | 0.9349 | 1.0541 |
|  | Female | 1.0132 | 0.9399 | 1.0923 |  | 0.8242 | 0.7615 | 0.8920 |  | 0.8134 | 0.7126 | 0.9285 |
| Suicide by suffocation | Both sexes | 0.9339 | 0.8957 | 0.9737 |  | 0.8904 | 0.8521 | 0.9304 |  | 0.9534 | 0.8854 | 1.0267 |
|  | Male | 0.9488 | 0.9049 | 0.9949 |  | 0.9023 | 0.8587 | 0.9481 |  | 0.9509 | 0.8745 | 1.0340 |
|  | Female | 0.9326 | 0.8538 | 1.0187 |  | 0.8298 | 0.7549 | 0.9122 |  | 0.8897 | 0.7600 | 1.0417 |

Notes: MtRR= mortality rate ratio, RMtRR=ratio of mortality rate ratio.

**Supplementary Table 5.** Ratio of age-standardized injury mortality rate ratios (RMtRR) from 2018 to 2019 and from 2019 to 2020, Americans aged 65 years and older.

| Cause of injury | Sex | From 2018 to 2019 | | |  | From 2019 to 2020 | | |  | 2019-2020 vs. 2018-2019 | | |
| --- | --- | --- | --- | --- | --- | --- | --- | --- | --- | --- | --- | --- |
|  |  | MtRR | 95% confidence interval | |  | MtRR | 95% confidence interval | |  | RMtRR | 95% confidence interval | |
| All injuries | Both sexes | 1.0234 | 1.0129 | 1.0341 |  | 1.0080 | 0.9979 | 1.0183 |  | 0.9849 | 0.9676 | 1.0025 |
|  | Male | 1.0282 | 1.0146 | 1.0421 |  | 1.0095 | 0.9965 | 1.0228 |  | 0.9818 | 0.9596 | 1.0045 |
|  | Female | 1.0172 | 1.0008 | 1.0339 |  | 1.0037 | 0.9878 | 1.0198 |  | 0.9867 | 0.9596 | 1.0145 |
| Unintentional injuries | Both sexes | 1.0332 | 1.0216 | 1.0449 |  | 1.0120 | 1.0010 | 1.0232 |  | 0.9795 | 0.9608 | 0.9985 |
|  | Male | 1.0436 | 1.0281 | 1.0594 |  | 1.0136 | 0.9990 | 1.0285 |  | 0.9713 | 0.9467 | 0.9964 |
|  | Female | 1.0205 | 1.0034 | 1.0379 |  | 1.0072 | 0.9907 | 1.0240 |  | 0.9870 | 0.9588 | 1.0160 |
| Motor vehicle traffic | Both sexes | 1.0139 | 0.9829 | 1.0458 |  | 0.9031 | 0.8754 | 0.9318 |  | 0.8908 | 0.8443 | 0.9398 |
|  | Male | 1.0308 | 0.9922 | 1.0710 |  | 0.9256 | 0.8910 | 0.9615 |  | 0.8979 | 0.8407 | 0.9589 |
|  | Female | 0.9888 | 0.9384 | 1.0419 |  | 0.8505 | 0.8059 | 0.8976 |  | 1.0265 | 0.9897 | 1.0647 |
| Occupant | Both sexes | 1.0221 | 0.9610 | 1.0870 |  | 0.7903 | 0.7411 | 0.8427 |  | 0.7732 | 0.6942 | 0.8612 |
|  | Male | 1.0660 | 0.9861 | 1.1524 |  | 0.8145 | 0.7522 | 0.8819 |  | 0.7640 | 0.6676 | 0.8744 |
|  | Female | 0.9688 | 0.8772 | 1.0700 |  | 0.7636 | 0.6868 | 0.8489 |  | 0.7882 | 0.6610 | 0.9398 |
| Motorcyclist | Both sexes | 1.0140 | 0.8926 | 1.1519 |  | 1.0657 | 0.9421 | 1.2056 |  | 1.0510 | 0.8455 | 1.3066 |
|  | Male | 1.0704 | 0.9361 | 1.2240 |  | 1.0453 | 0.9195 | 1.1884 |  | 0.9766 | 0.7783 | 1.2254 |
|  | Female | 0.6843 | 0.4322 | 1.0836 |  | 0.8469 | 0.5055 | 1.4187 |  | 1.2375 | 0.5280 | 2.9000 |
| Bicyclist | Both sexes | 1.0469 | 0.8216 | 1.3340 |  | 1.0926 | 0.8673 | 1.3765 |  | 1.0437 | 0.6922 | 1.5736 |
|  | Male | 1.3065 | 1.0126 | 1.6857 |  | 0.9769 | 0.7725 | 1.2353 |  | 0.7477 | 0.4919 | 1.1365 |
|  | Female | — | — | — |  | — | — | — |  | — | — | — |
| Pedestrian | Both sexes | 0.9745 | 0.9068 | 1.0472 |  | 0.9131 | 0.8488 | 0.9823 |  | 0.9370 | 0.8267 | 1.0620 |
|  | Male | 0.9908 | 0.9073 | 1.0820 |  | 0.8993 | 0.8225 | 0.9832 |  | 0.9076 | 0.7790 | 1.0575 |
|  | Female | 0.9338 | 0.8256 | 1.0561 |  | 0.9312 | 0.8212 | 1.0560 |  | 0.9973 | 0.8040 | 1.2371 |
| Falls | Both sexes | 1.0308 | 1.0156 | 1.0462 |  | 1.0457 | 1.0308 | 1.0608 |  | 1.0145 | 0.9891 | 1.0405 |
|  | Male | 1.0394 | 1.0183 | 1.0610 |  | 1.0421 | 1.0217 | 1.0630 |  | 1.0026 | 0.9682 | 1.0382 |
|  | Female | 1.0225 | 1.0009 | 1.0446 |  | 1.0496 | 1.0281 | 1.0716 |  | 1.0265 | 0.9897 | 1.0647 |
| Poisoning | Both sexes | 1.0991 | 1.0473 | 1.1535 |  | 1.1976 | 1.1455 | 1.2521 |  | 1.0896 | 1.0053 | 1.1811 |
|  | Male | 1.1347 | 1.0682 | 1.2053 |  | 1.2305 | 1.1649 | 1.2998 |  | 1.0844 | 0.9812 | 1.1985 |
|  | Female | 1.0503 | 0.9670 | 1.1407 |  | 1.1259 | 1.0412 | 1.2175 |  | 1.0720 | 0.9323 | 1.2327 |
| Drug poisoning | Both sexes | 1.1159 | 1.0595 | 1.1753 |  | 1.2080 | 1.1520 | 1.2668 |  | 1.0825 | 0.9930 | 1.1801 |
|  | Male | 1.1374 | 1.0659 | 1.2137 |  | 1.2557 | 1.1842 | 1.3315 |  | 1.1040 | 0.9916 | 1.2291 |
|  | Female | 1.0687 | 0.9787 | 1.1669 |  | 1.1121 | 1.0235 | 1.2085 |  | 1.0407 | 0.8972 | 1.2071 |
| Homicide | Both sexes | 0.9132 | 0.8393 | 0.9936 |  | 1.1076 | 1.0194 | 1.2034 |  | 1.2129 | 1.0480 | 1.4037 |
|  | Male | 0.8867 | 0.7937 | 0.9906 |  | 1.1318 | 1.0148 | 1.2623 |  | 1.2764 | 1.0530 | 1.5472 |
|  | Female | 0.8728 | 0.7667 | 0.9936 |  | 1.0933 | 0.9607 | 1.2442 |  | 1.2526 | 0.9989 | 1.5708 |
| Homicide by firearm | Both sexes | 0.9286 | 0.8160 | 1.0568 |  | 1.1763 | 1.0382 | 1.3327 |  | 1.2667 | 1.0143 | 1.5818 |
|  | Male | 0.9219 | 0.7776 | 1.0928 |  | 1.1384 | 0.9645 | 1.3436 |  | 1.2349 | 0.9209 | 1.6560 |
|  | Female | 0.8797 | 0.7237 | 1.0694 |  | 1.1850 | 0.9791 | 1.4342 |  | 1.3471 | 0.9603 | 1.8897 |
| Suicide | Both sexes | 0.9811 | 0.9532 | 1.0098 |  | 0.9711 | 0.9436 | 0.9995 |  | 0.9898 | 0.9417 | 1.0405 |
|  | Male | 0.9785 | 0.9484 | 1.0096 |  | 0.9819 | 0.9519 | 1.0129 |  | 1.0035 | 0.9508 | 1.0591 |
|  | Female | 0.9997 | 0.9313 | 1.0732 |  | 0.9303 | 0.8664 | 0.9989 |  | 0.9306 | 0.8231 | 1.0520 |
| Suicide by firearm | Both sexes | 0.9747 | 0.9418 | 1.0088 |  | 0.9857 | 0.9525 | 1.0200 |  | 1.0113 | 0.9530 | 1.0732 |
|  | Male | 0.9767 | 0.9427 | 1.0120 |  | 0.9901 | 0.9559 | 1.0256 |  | 1.0137 | 0.9535 | 1.0778 |
|  | Female | 0.9854 | 0.8761 | 1.1085 |  | 0.9514 | 0.8456 | 1.0705 |  | 0.9655 | 0.7876 | 1.1836 |
| Suicide by suffocation | Both sexes | 0.9895 | 0.9064 | 1.0802 |  | 0.9635 | 0.8829 | 1.0516 |  | 0.9737 | 0.8369 | 1.1330 |
|  | Male | 0.9657 | 0.8751 | 1.0656 |  | 0.9466 | 0.8572 | 1.0453 |  | 0.9802 | 0.8262 | 1.1631 |
|  | Female | 1.0508 | 0.8748 | 1.2622 |  | 0.9519 | 0.7946 | 1.1403 |  | 0.9059 | 0.6619 | 1.2398 |

Notes: MtRR= mortality rate ratio, RMtRR=ratio of mortality rate ratio.

—: Results were omitted for categories having unstable injury mortality rates (with 20 deaths or less).

**Supplementary Table 6.** Ratio of age-standardized injury morbidity rate ratios (RMbRR) from 2018 to 2019 and from 2019 to 2020, all Americans.

| Cause of injury | Sex | From 2018 to 2019 | | |  | From 2019 to 2020 | | |  | 2019-2020 vs. 2018-2019 | | |
| --- | --- | --- | --- | --- | --- | --- | --- | --- | --- | --- | --- | --- |
|  |  | MbRR | 95% confidence interval | |  | MbRR | 95% confidence interval | |  | RMbRR | 95% confidence interval | |
| All injuries | Both sexes | 0.9537 | 0.9532 | 0.9541 |  | 0.8437 | 0.8432 | 0.8441 |  | 0.8846 | 0.8838 | 0.8855 |
|  | Male | 0.9599 | 0.9593 | 0.9606 |  | 0.8672 | 0.8666 | 0.8679 |  | 0.9035 | 0.9023 | 0.9046 |
|  | Female | 0.9458 | 0.9450 | 0.9465 |  | 0.8158 | 0.8151 | 0.8164 |  | 0.8626 | 0.8613 | 0.8638 |
| Unintentional injuries | Both sexes | 0.9509 | 0.9504 | 0.9514 |  | 0.8345 | 0.8340 | 0.8349 |  | 0.8775 | 0.8767 | 0.8784 |
|  | Male | 0.9574 | 0.9567 | 0.9581 |  | 0.8589 | 0.8582 | 0.8595 |  | 0.8971 | 0.8959 | 0.8983 |
|  | Female | 0.9428 | 0.9421 | 0.9436 |  | 0.8055 | 0.8048 | 0.8062 |  | 0.8544 | 0.8531 | 0.8556 |
| Motor vehicle traffic | Both sexes | 0.9563 | 0.9548 | 0.9577 |  | 0.8739 | 0.8725 | 0.8753 |  | 0.9139 | 0.9115 | 0.9163 |
|  | Male | 0.9792 | 0.9772 | 0.9813 |  | 0.9255 | 0.9235 | 0.9274 |  | 0.9451 | 0.9416 | 0.9486 |
|  | Female | 0.9307 | 0.9287 | 0.9327 |  | 0.8162 | 0.8143 | 0.8181 |  | 0.8770 | 0.8736 | 0.8804 |
| Occupant | Both sexes | 0.9232 | 0.9215 | 0.9250 |  | 0.8053 | 0.8037 | 0.8070 |  | 0.8723 | 0.8694 | 0.8752 |
|  | Male | 0.9438 | 0.9412 | 0.9465 |  | 0.8475 | 0.8451 | 0.8500 |  | 0.8980 | 0.8935 | 0.9024 |
|  | Female | 0.9054 | 0.9031 | 0.9076 |  | 0.7700 | 0.7678 | 0.7721 |  | 0.8504 | 0.8466 | 0.8543 |
| Motorcyclist | Both sexes | 1.0464 | 1.0400 | 1.0528 |  | 1.1984 | 1.1915 | 1.2053 |  | 1.1453 | 1.1334 | 1.1572 |
|  | Male | 1.0497 | 1.0428 | 1.0567 |  | 1.1747 | 1.1673 | 1.1821 |  | 1.1190 | 1.1065 | 1.1317 |
|  | Female | 1.0315 | 1.0145 | 1.0488 |  | 1.3343 | 1.3139 | 1.3550 |  | 1.2935 | 1.2578 | 1.3302 |
| Bicyclist | Both sexes | 1.0682 | 1.0630 | 1.0734 |  | 1.0820 | 1.0769 | 1.0870 |  | 1.0129 | 1.0046 | 1.0213 |
|  | Male | 1.0733 | 1.0673 | 1.0794 |  | 1.0302 | 1.0246 | 1.0359 |  | 0.9598 | 0.9506 | 0.9691 |
|  | Female | 1.0495 | 1.0394 | 1.0597 |  | 1.2471 | 1.2358 | 1.2584 |  | 1.1882 | 1.1690 | 1.2078 |
| Pedestrian | Both sexes | 0.9749 | 0.9684 | 0.9814 |  | 0.7653 | 0.7599 | 0.7709 |  | 0.7851 | 0.7758 | 0.7945 |
|  | Male | 0.9512 | 0.9429 | 0.9597 |  | 0.8233 | 0.8156 | 0.8311 |  | 0.8655 | 0.8521 | 0.8792 |
|  | Female | 1.0065 | 0.9963 | 1.0168 |  | 0.6928 | 0.6851 | 0.7006 |  | 0.6883 | 0.6759 | 0.7010 |
| Falls | Both sexes | 0.9714 | 0.9704 | 0.9724 |  | 0.8340 | 0.8331 | 0.8349 |  | 0.8585 | 0.8570 | 0.8601 |
|  | Male | 0.9782 | 0.9768 | 0.9797 |  | 0.8492 | 0.8479 | 0.8505 |  | 0.8681 | 0.8658 | 0.8703 |
|  | Female | 0.9644 | 0.9631 | 0.9657 |  | 0.8224 | 0.8212 | 0.8236 |  | 0.8528 | 0.8507 | 0.8549 |
| Poisoning | Both sexes | 1.0243 | 1.0222 | 1.0265 |  | 1.0280 | 1.0258 | 1.0301 |  | 1.0035 | 0.9999 | 1.0072 |
|  | Male | 1.0264 | 1.0238 | 1.0291 |  | 1.0406 | 1.0380 | 1.0433 |  | 1.0138 | 1.0093 | 1.0184 |
|  | Female | 1.0200 | 1.0164 | 1.0237 |  | 1.0024 | 0.9988 | 1.0059 |  | 0.9827 | 0.9767 | 0.9888 |
| Assault | Both sexes | 0.9788 | 0.9766 | 0.9809 |  | 0.9350 | 0.9329 | 0.9371 |  | 0.9553 | 0.9516 | 0.9590 |
|  | Male | 0.9882 | 0.9854 | 0.9911 |  | 0.9583 | 0.9555 | 0.9611 |  | 0.9697 | 0.9648 | 0.9745 |
|  | Female | 0.9641 | 0.9609 | 0.9674 |  | 0.9018 | 0.8986 | 0.9050 |  | 0.9354 | 0.9298 | 0.9410 |
| Self-harm | Both sexes | 1.0001 | 0.9963 | 1.0040 |  | 0.9838 | 0.9800 | 0.9876 |  | 0.9836 | 0.9771 | 0.9902 |
|  | Male | 0.9715 | 0.9656 | 0.9774 |  | 0.9824 | 0.9764 | 0.9885 |  | 1.0112 | 1.0005 | 1.0220 |
|  | Female | 1.0198 | 1.0148 | 1.0249 |  | 0.9864 | 0.9815 | 0.9912 |  | 0.9672 | 0.9590 | 0.9755 |
| Self-harm by suffocation | Both sexes | 1.1872 | 1.1505 | 1.2251 |  | 0.7808 | 0.7562 | 0.8062 |  | 0.6577 | 0.6231 | 0.6941 |
|  | Male | 1.1719 | 1.1271 | 1.2184 |  | 0.7771 | 0.7467 | 0.8087 |  | 0.6631 | 0.6201 | 0.7092 |
|  | Female | 1.2206 | 1.1566 | 1.2881 |  | 0.7853 | 0.7437 | 0.8292 |  | 0.6434 | 0.5868 | 0.7053 |

Notes: MbRR = morbidity rate ratio, RMbRR =ratio of morbidity rate ratio.

**Supplementary Table 7.** Ratio of age-standardized injury morbidity rate ratios (RMbRR) from 2018 to 2019 and from 2019 to 2020, Americans aged 0-24 years old.

| Cause of injury | Sex | From 2018 to 2019 | | |  | From 2019 to 2020 | | |  | 2019-2020 vs. 2018-2019 | | |
| --- | --- | --- | --- | --- | --- | --- | --- | --- | --- | --- | --- | --- |
|  |  | MbRR | 95% confidence interval | |  | MbRR | 95% confidence interval | |  | RMbRR | 95% confidence interval | |
| All injuries | Both sexes | 0.9483 | 0.9474 | 0.9492 |  | 0.7789 | 0.7781 | 0.7797 |  | 0.8213 | 0.8199 | 0.8228 |
|  | Male | 0.9564 | 0.9552 | 0.9575 |  | 0.7732 | 0.7722 | 0.7743 |  | 0.8085 | 0.8067 | 0.8104 |
|  | Female | 0.9383 | 0.9370 | 0.9396 |  | 0.7861 | 0.7849 | 0.7873 |  | 0.8378 | 0.8356 | 0.8400 |
| Unintentional injuries | Both sexes | 0.9445 | 0.9436 | 0.9454 |  | 0.7676 | 0.7668 | 0.7684 |  | 0.8127 | 0.8112 | 0.8142 |
|  | Male | 0.9538 | 0.9526 | 0.9551 |  | 0.7674 | 0.7663 | 0.7685 |  | 0.8045 | 0.8026 | 0.8065 |
|  | Female | 0.9326 | 0.9313 | 0.9340 |  | 0.7679 | 0.7666 | 0.7691 |  | 0.8233 | 0.8211 | 0.8256 |
| Motor vehicle traffic | Both sexes | 0.9480 | 0.9454 | 0.9506 |  | 0.9105 | 0.9079 | 0.9132 |  | 0.9605 | 0.9558 | 0.9652 |
|  | Male | 0.9813 | 0.9775 | 0.9851 |  | 0.9386 | 0.9349 | 0.9422 |  | 0.9564 | 0.9500 | 0.9629 |
|  | Female | 0.9124 | 0.9088 | 0.9161 |  | 0.8782 | 0.8745 | 0.8820 |  | 0.9625 | 0.9556 | 0.9695 |
| Occupant | Both sexes | 0.9079 | 0.9047 | 0.9112 |  | 0.8495 | 0.8462 | 0.8528 |  | 0.9357 | 0.9297 | 0.9417 |
|  | Male | 0.9405 | 0.9355 | 0.9457 |  | 0.8753 | 0.8703 | 0.8803 |  | 0.9306 | 0.9217 | 0.9397 |
|  | Female | 0.8832 | 0.8790 | 0.8874 |  | 0.8285 | 0.8242 | 0.8328 |  | 0.9380 | 0.9300 | 0.9462 |
| Motorcyclist | Both sexes | 1.0164 | 1.0045 | 1.0284 |  | 1.1812 | 1.1679 | 1.1946 |  | 1.1621 | 1.1390 | 1.1857 |
|  | Male | 1.0211 | 1.0082 | 1.0341 |  | 1.1747 | 1.1605 | 1.1890 |  | 1.1504 | 1.1259 | 1.1755 |
|  | Female | 0.9910 | 0.9597 | 1.0235 |  | 1.2271 | 1.1899 | 1.2655 |  | 1.2382 | 1.1719 | 1.3082 |
| Bicyclist | Both sexes | 1.0636 | 1.0558 | 1.0714 |  | 1.0870 | 1.0793 | 1.0948 |  | 1.0221 | 1.0093 | 1.0350 |
|  | Male | 1.0923 | 1.0830 | 1.1016 |  | 1.0099 | 1.0015 | 1.0183 |  | 0.9246 | 0.9112 | 0.9381 |
|  | Female | 0.9834 | 0.9691 | 0.9979 |  | 1.3278 | 1.3096 | 1.3462 |  | 1.3502 | 1.3169 | 1.3843 |
| Pedestrian | Both sexes | 0.9484 | 0.9361 | 0.9609 |  | 0.7050 | 0.6948 | 0.7154 |  | 0.7433 | 0.7260 | 0.7611 |
|  | Male | 0.9463 | 0.9301 | 0.9627 |  | 0.6729 | 0.6600 | 0.6862 |  | 0.7112 | 0.6892 | 0.7338 |
|  | Female | 0.9511 | 0.9321 | 0.9704 |  | 0.7491 | 0.7328 | 0.7659 |  | 0.7877 | 0.7597 | 0.8167 |
| Falls | Both sexes | 0.9684 | 0.9666 | 0.9702 |  | 0.7616 | 0.7600 | 0.7631 |  | 0.7864 | 0.7838 | 0.7891 |
|  | Male | 0.9836 | 0.9811 | 0.9861 |  | 0.7597 | 0.7577 | 0.7618 |  | 0.7724 | 0.7689 | 0.7759 |
|  | Female | 0.9497 | 0.9470 | 0.9523 |  | 0.7639 | 0.7615 | 0.7662 |  | 0.8044 | 0.8003 | 0.8085 |
| Poisoning | Both sexes | 0.9887 | 0.9834 | 0.9940 |  | 0.9831 | 0.9778 | 0.9885 |  | 0.9944 | 0.9852 | 1.0037 |
|  | Male | 0.9585 | 0.9517 | 0.9655 |  | 0.9824 | 0.9752 | 0.9896 |  | 1.0249 | 1.0120 | 1.0379 |
|  | Female | 1.0281 | 1.0200 | 1.0364 |  | 0.9844 | 0.9766 | 0.9923 |  | 0.9574 | 0.9443 | 0.9708 |
| Assault | Both sexes | 0.9660 | 0.9620 | 0.9699 |  | 0.8410 | 0.8374 | 0.8447 |  | 0.8707 | 0.8644 | 0.8770 |
|  | Male | 0.9783 | 0.9729 | 0.9837 |  | 0.8151 | 0.8103 | 0.8199 |  | 0.8332 | 0.8251 | 0.8413 |
|  | Female | 0.9513 | 0.9455 | 0.9571 |  | 0.8733 | 0.8677 | 0.8789 |  | 0.9180 | 0.9081 | 0.9279 |
| Self-harm | Both sexes | 1.0466 | 1.0403 | 1.0530 |  | 1.0328 | 1.0267 | 1.0390 |  | 0.9869 | 0.9766 | 0.9972 |
|  | Male | 1.0199 | 1.0087 | 1.0311 |  | 0.9884 | 0.9776 | 0.9993 |  | 0.9691 | 0.9508 | 0.9877 |
|  | Female | 1.0581 | 1.0505 | 1.0659 |  | 1.0509 | 1.0435 | 1.0584 |  | 0.9932 | 0.9809 | 1.0056 |
| Self-harm by suffocation | Both sexes | — | — | — |  | — | — | — |  | — | — | — |
|  | Male | — | — | — |  | — | — | — |  | — | — | — |
|  | Female | — | — | — |  | — | — | — |  | — | — | — |

Notes: MbRR = morbidity rate ratio, RMbRR =ratio of morbidity rate ratio.

—: Results were omitted for categories having unstable injury morbidity rates (due to 20 injuries or less, the national estimates less than 1,200, the coefficient of variation greater than 30%, or the tool not involving details regarding mechanism of relevant injury).

**Supplementary Table 8.** Ratio of age-standardized injury morbidity rate ratios (RMbRR) from 2018 to 2019 and from 2019 to 2020, Americans aged 25-44 years old.

| Cause of injury | Sex | From 2018 to 2019 | | |  | From 2019 to 2020 | | |  | 2019-2020 vs. 2018-2019 | | |
| --- | --- | --- | --- | --- | --- | --- | --- | --- | --- | --- | --- | --- |
|  |  | MbRR | 95% confidence interval | |  | MbRR | 95% confidence interval | |  | RMbRR | 95% confidence interval | |
| All injuries | Both sexes | 0.9392 | 0.9383 | 0.9401 |  | 0.8867 | 0.8858 | 0.8875 |  | 0.9440 | 0.9424 | 0.9457 |
|  | Male | 0.9456 | 0.9445 | 0.9468 |  | 0.9193 | 0.9181 | 0.9205 |  | 0.9721 | 0.9699 | 0.9744 |
|  | Female | 0.9302 | 0.9288 | 0.9315 |  | 0.8404 | 0.8391 | 0.8417 |  | 0.9035 | 0.9011 | 0.9060 |
| Unintentional injuries | Both sexes | 0.9346 | 0.9336 | 0.9355 |  | 0.8751 | 0.8742 | 0.8761 |  | 0.9364 | 0.9346 | 0.9381 |
|  | Male | 0.9415 | 0.9403 | 0.9428 |  | 0.9069 | 0.9057 | 0.9082 |  | 0.9633 | 0.9609 | 0.9656 |
|  | Female | 0.9250 | 0.9236 | 0.9265 |  | 0.8304 | 0.8290 | 0.8318 |  | 0.8977 | 0.8951 | 0.9003 |
| Motor vehicle traffic | Both sexes | 0.9531 | 0.9506 | 0.9555 |  | 0.8894 | 0.8870 | 0.8917 |  | 0.9331 | 0.9289 | 0.9374 |
|  | Male | 0.9701 | 0.9667 | 0.9735 |  | 0.9587 | 0.9552 | 0.9621 |  | 0.9882 | 0.9821 | 0.9944 |
|  | Female | 0.9344 | 0.9309 | 0.9379 |  | 0.8096 | 0.8063 | 0.8128 |  | 0.8664 | 0.8606 | 0.8722 |
| Occupant | Both sexes | 0.9358 | 0.9330 | 0.9386 |  | 0.8172 | 0.8146 | 0.8199 |  | 0.8733 | 0.8686 | 0.8780 |
|  | Male | 0.9550 | 0.9508 | 0.9592 |  | 0.8618 | 0.8579 | 0.8658 |  | 0.9024 | 0.8954 | 0.9095 |
|  | Female | 0.9193 | 0.9154 | 0.9231 |  | 0.7766 | 0.7731 | 0.7802 |  | 0.8448 | 0.8385 | 0.8512 |
| Motorcyclist | Both sexes | 1.0261 | 1.0159 | 1.0364 |  | 1.3284 | 1.3162 | 1.3408 |  | 1.2947 | 1.2731 | 1.3166 |
|  | Male | 1.0098 | 0.9991 | 1.0207 |  | 1.3253 | 1.3121 | 1.3385 |  | 1.3124 | 1.2889 | 1.3363 |
|  | Female | 1.1397 | 1.1086 | 1.1717 |  | 1.3414 | 1.3084 | 1.3753 |  | 1.1770 | 1.1242 | 1.2323 |
| Bicyclist | Both sexes | 1.0722 | 1.0610 | 1.0835 |  | 1.0851 | 1.0742 | 1.0961 |  | 1.0120 | 0.9941 | 1.0303 |
|  | Male | 1.0345 | 1.0222 | 1.0469 |  | 1.0849 | 1.0724 | 1.0975 |  | 1.0487 | 1.0276 | 1.0703 |
|  | Female | 1.2130 | 1.1863 | 1.2404 |  | 1.0870 | 1.0647 | 1.1098 |  | 0.8961 | 0.8635 | 0.9300 |
| Pedestrian | Both sexes | 0.9411 | 0.9302 | 0.9522 |  | 0.8222 | 0.8121 | 0.8325 |  | 0.8737 | 0.8557 | 0.8920 |
|  | Male | 0.9339 | 0.9197 | 0.9484 |  | 0.9170 | 0.9025 | 0.9317 |  | 0.9819 | 0.9557 | 1.0089 |
|  | Female | 0.9508 | 0.9339 | 0.9680 |  | 0.6941 | 0.6804 | 0.7081 |  | 0.7300 | 0.7067 | 0.7541 |
| Falls | Both sexes | 0.9200 | 0.9176 | 0.9223 |  | 0.8328 | 0.8305 | 0.8351 |  | 0.9053 | 0.9011 | 0.9094 |
|  | Male | 0.9372 | 0.9337 | 0.9406 |  | 0.8433 | 0.8401 | 0.8466 |  | 0.8999 | 0.8940 | 0.9058 |
|  | Female | 0.9041 | 0.9009 | 0.9074 |  | 0.8226 | 0.8194 | 0.8257 |  | 0.9098 | 0.9039 | 0.9157 |
| Poisoning | Both sexes | 1.0306 | 1.0272 | 1.0339 |  | 1.0588 | 1.0554 | 1.0621 |  | 1.0274 | 1.0217 | 1.0331 |
|  | Male | 1.0442 | 1.0401 | 1.0483 |  | 1.0590 | 1.0550 | 1.0631 |  | 1.0142 | 1.0074 | 1.0211 |
|  | Female | 1.0029 | 0.9972 | 1.0086 |  | 1.0565 | 1.0506 | 1.0624 |  | 1.0535 | 1.0431 | 1.0639 |
| Assault | Both sexes | 0.9766 | 0.9734 | 0.9799 |  | 0.9712 | 0.9679 | 0.9745 |  | 0.9944 | 0.9886 | 1.0002 |
|  | Male | 0.9866 | 0.9824 | 0.9908 |  | 1.0085 | 1.0043 | 1.0129 |  | 1.0222 | 1.0147 | 1.0299 |
|  | Female | 0.9604 | 0.9553 | 0.9655 |  | 0.9127 | 0.9077 | 0.9177 |  | 0.9503 | 0.9415 | 0.9592 |
| Self-harm | Both sexes | 0.9527 | 0.9463 | 0.9591 |  | 0.9573 | 0.9507 | 0.9639 |  | 1.0049 | 0.9931 | 1.0168 |
|  | Male | 0.8798 | 0.8713 | 0.8883 |  | 1.0202 | 1.0102 | 1.0304 |  | 1.1597 | 1.1401 | 1.1796 |
|  | Female | 1.0260 | 1.0164 | 1.0357 |  | 0.9027 | 0.8942 | 0.9114 |  | 0.8799 | 0.8656 | 0.8944 |
| Self-harm by suffocation | Both sexes | 0.6816 | 0.6489 | 0.7159 |  | 1.0818 | 1.0266 | 1.1400 |  | 1.5872 | 1.4512 | 1.7360 |
|  | Male | 0.6783 | 0.6409 | 0.7179 |  | 1.1029 | 1.0384 | 1.1714 |  | 1.6260 | 1.4664 | 1.8029 |
|  | Female | — | — | — |  | — | — | — |  | — | — | — |

Notes: MbRR = morbidity rate ratio, RMbRR =ratio of morbidity rate ratio.

—: Results were omitted for categories having unstable injury morbidity rates (due to 20 injuries or less, the national estimates less than 1,200, the coefficient of variation greater than 30%, or the tool not involving details regarding mechanism of relevant injury).

**Supplementary Table 9.** Ratio of age-standardized injury morbidity rate ratios (RMbRR) from 2018 to 2019 and from 2019 to 2020, Americans aged 45-64 years old.

| Cause of injury | Sex | From 2018 to 2019 | | |  | From 2019 to 2020 | | |  | 2019-2020 vs. 2018-2019 | | |
| --- | --- | --- | --- | --- | --- | --- | --- | --- | --- | --- | --- | --- |
|  |  | MbRR | 95% confidence interval | |  | MbRR | 95% confidence interval | |  | RMbRR | 95% confidence interval | |
| All injuries | Both sexes | 0.9480 | 0.9470 | 0.9490 |  | 0.8729 | 0.8719 | 0.8738 |  | 0.9208 | 0.9190 | 0.9225 |
|  | Male | 0.9587 | 0.9574 | 0.9600 |  | 0.9161 | 0.9147 | 0.9174 |  | 0.9555 | 0.9531 | 0.9580 |
|  | Female | 0.9342 | 0.9327 | 0.9357 |  | 0.8162 | 0.8148 | 0.8177 |  | 0.8738 | 0.8712 | 0.8763 |
| Unintentional injuries | Both sexes | 0.9435 | 0.9425 | 0.9445 |  | 0.8662 | 0.8652 | 0.8672 |  | 0.9181 | 0.9163 | 0.9199 |
|  | Male | 0.9526 | 0.9512 | 0.9539 |  | 0.9099 | 0.9085 | 0.9113 |  | 0.9552 | 0.9526 | 0.9577 |
|  | Female | 0.9320 | 0.9304 | 0.9335 |  | 0.8099 | 0.8085 | 0.8114 |  | 0.8691 | 0.8664 | 0.8717 |
| Motor vehicle traffic | Both sexes | 0.9523 | 0.9494 | 0.9552 |  | 0.8163 | 0.8136 | 0.8189 |  | 0.8571 | 0.8524 | 0.8619 |
|  | Male | 0.9807 | 0.9766 | 0.9849 |  | 0.8643 | 0.8605 | 0.8681 |  | 0.8813 | 0.8747 | 0.8879 |
|  | Female | 0.9207 | 0.9166 | 0.9248 |  | 0.7607 | 0.7570 | 0.7645 |  | 0.8262 | 0.8195 | 0.8329 |
| Occupant | Both sexes | 0.9156 | 0.9121 | 0.9191 |  | 0.7519 | 0.7488 | 0.7551 |  | 0.8212 | 0.8155 | 0.8269 |
|  | Male | 0.9370 | 0.9317 | 0.9423 |  | 0.8087 | 0.8038 | 0.8136 |  | 0.8630 | 0.8544 | 0.8718 |
|  | Female | 0.8973 | 0.8927 | 0.9020 |  | 0.7025 | 0.6984 | 0.7067 |  | 0.7829 | 0.7755 | 0.7904 |
| Motorcyclist | Both sexes | 1.0952 | 1.0824 | 1.1082 |  | 1.0302 | 1.0185 | 1.0421 |  | 0.9407 | 0.9220 | 0.9597 |
|  | Male | 1.1359 | 1.1215 | 1.1505 |  | 0.9616 | 0.9497 | 0.9737 |  | 0.8466 | 0.8284 | 0.8652 |
|  | Female | 0.8836 | 0.8567 | 0.9113 |  | 1.4907 | 1.4478 | 1.5349 |  | 1.6871 | 1.5997 | 1.7793 |
| Bicyclist | Both sexes | 1.0645 | 1.0539 | 1.0753 |  | 0.9195 | 0.9102 | 0.9289 |  | 0.8638 | 0.8489 | 0.8789 |
|  | Male | 1.0686 | 1.0562 | 1.0811 |  | 0.8773 | 0.8669 | 0.8878 |  | 0.8209 | 0.8045 | 0.8377 |
|  | Female | 1.0498 | 1.0292 | 1.0709 |  | 1.0436 | 1.0235 | 1.0641 |  | 0.9940 | 0.9608 | 1.0284 |
| Pedestrian | Both sexes | 0.9824 | 0.9700 | 0.9948 |  | 0.7774 | 0.7669 | 0.7880 |  | 0.7913 | 0.7738 | 0.8093 |
|  | Male | 0.9183 | 0.9033 | 0.9335 |  | 0.8640 | 0.8490 | 0.8793 |  | 0.9409 | 0.9137 | 0.9689 |
|  | Female | 1.0811 | 1.0601 | 1.1026 |  | 0.6635 | 0.6493 | 0.6781 |  | 0.6137 | 0.5926 | 0.6356 |
| Falls | Both sexes | 0.9400 | 0.9380 | 0.9420 |  | 0.8605 | 0.8586 | 0.8625 |  | 0.9155 | 0.9120 | 0.9190 |
|  | Male | 0.9345 | 0.9315 | 0.9374 |  | 0.8996 | 0.8966 | 0.9025 |  | 0.9626 | 0.9573 | 0.9681 |
|  | Female | 0.9448 | 0.9421 | 0.9475 |  | 0.8286 | 0.8260 | 0.8311 |  | 0.8770 | 0.8725 | 0.8815 |
| Poisoning | Both sexes | 1.0400 | 1.0363 | 1.0437 |  | 1.0242 | 1.0206 | 1.0278 |  | 0.9848 | 0.9788 | 0.9909 |
|  | Male | 1.0363 | 1.0319 | 1.0408 |  | 1.0539 | 1.0495 | 1.0583 |  | 1.0169 | 1.0095 | 1.0244 |
|  | Female | 1.0499 | 1.0430 | 1.0569 |  | 0.9511 | 0.9448 | 0.9574 |  | 0.9058 | 0.8955 | 0.9163 |
| Assault | Both sexes | 1.0179 | 1.0129 | 1.0230 |  | 0.9842 | 0.9794 | 0.9891 |  | 0.9669 | 0.9587 | 0.9752 |
|  | Male | 1.0205 | 1.0143 | 1.0268 |  | 1.0257 | 1.0195 | 1.0320 |  | 1.0051 | 0.9944 | 1.0158 |
|  | Female | 1.0127 | 1.0044 | 1.0211 |  | 0.9091 | 0.9014 | 0.9168 |  | 0.8977 | 0.8848 | 0.9107 |
| Self-harm | Both sexes | 1.0069 | 0.9979 | 1.0160 |  | 0.9113 | 0.9029 | 0.9197 |  | 0.9050 | 0.8909 | 0.9193 |
|  | Male | 1.1834 | 1.1682 | 1.1988 |  | 0.8669 | 0.8558 | 0.8781 |  | 0.7325 | 0.7166 | 0.7489 |
|  | Female | 0.8611 | 0.8503 | 0.8720 |  | 0.9633 | 0.9507 | 0.9761 |  | 1.1187 | 1.0939 | 1.1441 |
| Self-harm by suffocation | Both sexes | — | — | — |  | — | — | — |  | — | — | — |
|  | Male | — | — | — |  | — | — | — |  | — | — | — |
|  | Female | — | — | — |  | — | — | — |  | — | — | — |

Notes: MbRR = morbidity rate ratio, RMbRR =ratio of morbidity rate ratio.

—: Results were omitted for categories having unstable injury morbidity rates (due to 20 injuries or less, the national estimates less than 1,200, the coefficient of variation greater than 30%, or the tool not involving details regarding mechanism of relevant injury).

**Supplementary Table 10.** Ratio of age-standardized injury morbidity rate ratios (RMbRR) from 2018 to 2019 and from 2019 to 2020, Americans aged 65 years and older.

| Cause of injury | Sex | From 2018 to 2019 | | |  | From 2019 to 2020 | | |  | 2019-2020 vs. 2018-2019 | | |
| --- | --- | --- | --- | --- | --- | --- | --- | --- | --- | --- | --- | --- |
|  |  | MbRR | 95% confidence interval | |  | MbRR | 95% confidence interval | |  | RMbRR | 95% confidence interval | |
| All injuries | Both sexes | 1.0099 | 1.0087 | 1.0111 |  | 0.8600 | 0.8590 | 0.8611 |  | 0.8516 | 0.8498 | 0.8535 |
|  | Male | 1.0131 | 1.0112 | 1.0150 |  | 0.8993 | 0.8976 | 0.9010 |  | 0.8877 | 0.8847 | 0.8906 |
|  | Female | 1.0070 | 1.0054 | 1.0086 |  | 0.8317 | 0.8304 | 0.8331 |  | 0.8259 | 0.8235 | 0.8283 |
| Unintentional injuries | Both sexes | 1.0111 | 1.0099 | 1.0123 |  | 0.8578 | 0.8567 | 0.8588 |  | 0.8484 | 0.8465 | 0.8502 |
|  | Male | 1.0159 | 1.0141 | 1.0178 |  | 0.8957 | 0.8940 | 0.8974 |  | 0.8817 | 0.8787 | 0.8846 |
|  | Female | 1.0071 | 1.0055 | 1.0087 |  | 0.8306 | 0.8292 | 0.8320 |  | 0.8247 | 0.8223 | 0.8271 |
| Motor vehicle traffic | Both sexes | 1.0144 | 1.0097 | 1.0190 |  | 0.8069 | 0.8031 | 0.8108 |  | 0.7955 | 0.7891 | 0.8019 |
|  | Male | 1.0087 | 1.0021 | 1.0153 |  | 0.8834 | 0.8776 | 0.8893 |  | 0.8758 | 0.8659 | 0.8859 |
|  | Female | 1.0162 | 1.0097 | 1.0226 |  | 0.7383 | 0.7333 | 0.7433 |  | 0.7265 | 0.7184 | 0.7348 |
| Occupant | Both sexes | 0.9366 | 0.9310 | 0.9423 |  | 0.7082 | 0.7035 | 0.7129 |  | 0.7561 | 0.7479 | 0.7644 |
|  | Male | 0.9142 | 0.9059 | 0.9226 |  | 0.7737 | 0.7661 | 0.7814 |  | 0.8463 | 0.8325 | 0.8603 |
|  | Female | 0.9520 | 0.9444 | 0.9597 |  | 0.6613 | 0.6554 | 0.6673 |  | 0.6946 | 0.6846 | 0.7049 |
| Motorcyclist | Both sexes | 1.2344 | 1.2004 | 1.2694 |  | 0.9855 | 0.9601 | 1.0116 |  | 0.7984 | 0.7622 | 0.8363 |
|  | Male | 1.2043 | 1.1686 | 1.2410 |  | 0.9642 | 0.9372 | 0.9920 |  | 0.8006 | 0.7615 | 0.8418 |
|  | Female | — | — | — |  | — | — | — |  | — | — | — |
| Bicyclist | Both sexes | — | — | — |  | — | — | — |  | — | — | — |
|  | Male | — | — | — |  | — | — | — |  | — | — | — |
|  | Female | — | — | — |  | — | — | — |  | — | — | — |
| Pedestrian | Both sexes | 1.1832 | 1.1611 | 1.2056 |  | 0.7088 | 0.6952 | 0.7227 |  | 0.5991 | 0.5800 | 0.6188 |
|  | Male | 1.1285 | 1.0997 | 1.1582 |  | 0.8133 | 0.7923 | 0.8348 |  | 0.7207 | 0.6893 | 0.7534 |
|  | Female | 1.2435 | 1.2100 | 1.2778 |  | 0.6046 | 0.5873 | 0.6225 |  | 0.4863 | 0.4637 | 0.5099 |
| Falls | Both sexes | 1.0223 | 1.0207 | 1.0238 |  | 0.8879 | 0.8865 | 0.8892 |  | 0.8685 | 0.8662 | 0.8709 |
|  | Male | 1.0266 | 1.0241 | 1.0291 |  | 0.9295 | 0.9273 | 0.9318 |  | 0.9055 | 0.9016 | 0.9094 |
|  | Female | 1.0193 | 1.0173 | 1.0212 |  | 0.8639 | 0.8622 | 0.8655 |  | 0.8475 | 0.8446 | 0.8504 |
| Poisoning | Both sexes | 1.0023 | 0.9947 | 1.0100 |  | 0.9302 | 0.9232 | 0.9373 |  | 0.9280 | 0.9159 | 0.9403 |
|  | Male | 0.9979 | 0.9880 | 1.0078 |  | 0.9504 | 0.9410 | 0.9599 |  | 0.9524 | 0.9362 | 0.9690 |
|  | Female | 0.9960 | 0.9842 | 1.0078 |  | 0.8977 | 0.8870 | 0.9086 |  | 0.9014 | 0.8830 | 0.9201 |
| Assault | Both sexes | 0.8986 | 0.8853 | 0.9121 |  | 1.1885 | 1.1714 | 1.2058 |  | 1.3227 | 1.2890 | 1.3572 |
|  | Male | 0.8760 | 0.8594 | 0.8930 |  | 1.1845 | 1.1626 | 1.2069 |  | 1.3522 | 1.3080 | 1.3978 |
|  | Female | 0.9290 | 0.9069 | 0.9516 |  | 1.1947 | 1.1673 | 1.2227 |  | 1.2860 | 1.2340 | 1.3403 |
| Self-harm | Both sexes | 0.8612 | 0.8429 | 0.8798 |  | 0.9206 | 0.9003 | 0.9414 |  | 1.0691 | 1.0293 | 1.1104 |
|  | Male | 0.6806 | 0.6596 | 0.7022 |  | 1.2500 | 1.2107 | 1.2906 |  | 1.8367 | 1.7364 | 1.9428 |
|  | Female | 1.0678 | 1.0366 | 1.0998 |  | 0.6931 | 0.6715 | 0.7154 |  | 0.6491 | 0.6161 | 0.6838 |
| Self-harm by suffocation | Both sexes | — | — | — |  | — | — | — |  | — | — | — |
|  | Male | — | — | — |  | — | — | — |  | — | — | — |
|  | Female | — | — | — |  | — | — | — |  | — | — | — |

Notes: MbRR = morbidity rate ratio, RMbRR =ratio of morbidity rate ratio.

—: Results were omitted for categories having unstable injury morbidity rates (due to 20 injuries or less, the national estimates less than 1,200, the coefficient of variation greater than 30%, or the tool not involving details regarding mechanism of relevant injury).
